# Redescription and Phylogenetic Placement of †*Hemicalypterus weiri* Schaeffer, 1967 (Actinopterygii, Neopterygii) from the Triassic Chinle Formation, Southwestern United States: New Insights into Morphology, Ecological Niche, and Phylogeny

**DOI:** 10.1371/journal.pone.0163657

**Published:** 2016-09-22

**Authors:** Sarah Z. Gibson

**Affiliations:** Department of Geology and Biodiversity Institute, University of Kansas, Lawrence, Kansas, United States of America; Laboratoire de Biologie du Développement de Villefranche-sur-Mer, FRANCE

## Abstract

The actinopterygian fish †*Hemicalypterus weiri* Schaeffer, 1967 is herein redescribed and rediagnosed based on new information collected from reexamination of museum specimens as well as examination of recently collected specimens from the Upper Triassic Chinle Formation of San Juan County, Utah, United States. †*Hemicalypterus* is distinguishable by its deep, disc-shaped compressed body; ganoid-scaled anterior half and scaleless posterior half; spinose, prominent dorsal and ventral ridge scales anterior to dorsal and anal fins; hem-like dorsal and anal fins with rounded distal margins; small mouth gape; and specialized, multicuspid dentition. This type of dentition, when observed in extant fishes, is often associated with herbivory, and †*Hemicalypterus* represents the oldest known ray-finned fish to have possibly exploited an herbivorous trophic feeding niche. A phylogenetic analysis infers a placement of †*Hemicalypterus* within †Dapediiformes, with †Dapediiformes being recovered as sister to Ginglymodi within holostean actinopterygians.

## Introduction

Ray-finned fishes (Actinopterygii) are one of the most successful groups of vertebrates on Earth, represented by over 30,000 species (e.g., [1, 2]). In addition, ray-finned fishes have evolved to occupy multiple ecological niches in both freshwater and marine habitats, and consequently have evolved a vast array of morphological variation associated with different habitats, diets, behaviors, and ecologies. The evolutionary history of ray-finned fishes is equally expansive, with representatives in the fossil record occurring clear into the Late Silurian (~420 million years ago) [3]. It has been long considered that ray-finned fishes were carnivorous, generalist feeders in the Paleozoic and most of the Mesozoic, with no anatomical fossil evidence of herbivory until the Eocene (~50 Ma) [4]. A recent study [5] on the Triassic deep-bodied neopterygian fish †*Hemicalypterus* Schaeffer, 1967 [6], provided morphological evidence of a specialized, multicuspid dentition that has never before been seen in a non-teleost ray-finned fish. This morphology is found within several extant fish lineages, such as surgeonfishes (e.g., *Acanthurus*, *Zebrasoma*) and rabbitfishes (e.g., *Siganus*), which both occupy marine coral reef habitats, as well as freshwater haplochromine cichlids (e.g., *Maylandia*, *Labeotropheus*) and characiforms (e.g., *Hyphressobrycon*) [5]. The modern fishes that display this specialized multicuspid dentition are known to be benthic feeders scraping the substratum, feeding on algae as a predominant food source [5]. The specialized, multicuspid dentition observed in †*Hemicalypterus* suggests that it occupied a benthic scraping, herbivorous trophic niche, and is the oldest potential evidence of herbivory in ray-finned fishes [5].

†*Hemicalypterus* is a monotypic genus whose evolutionary relationship to other actinopterygian fishes has previously been unclear. †*Hemicalypterus* was discovered in the mid-twentieth century by scientists from the United States Geological Survey and the American Museum of Natural History in southeastern Utah, United States, and was described by Schaeffer in 1967 [6]. Additional putative specimens of †*Hemicalypterus* (identified as cf. *Hemicalytperus* sp. in [7]) were recovered from the Redonda Formation of eastern New Mexico and are reported in Johnson et al. [7]. These specimens are preserved only as patches of scales and a possible “premaxilla” [7]. Due to the incompleteness of these specimens, it is difficult to attribute them to the species level, but Johnson et al. [7] stated that they are potentially †*Hemicalypterus weiri*. These specimens were reported in Milner et al. [8] as a distinct occurrence of †*Hemicalypterus weiri* in the Redonda Formation. Without further evidence that these fragmentary specimens are definitively †*H*. *weiri*, the geographic distribution of †*H*. *weiri* as discussed herein is restricted to the Chinle Formation of southeastern Utah.

The initial morphological study and description of †*Hemicalypterus weiri* by Schaeffer [6] did not identify the specialized, multidenticulate teeth (instead misidentifying a single, isolated tooth as a disarticulated premaxilla; [6]); however, these teeth were identified by Gibson [5]. Schaeffer’s [6] description was also based on a limited number of specimens, many lacking critical morphological information that could provide evidence of evolutionary relationships to other ray-finned fishes. However, because of certain skull features he recognized as being similar to other semionotid fishes, Schaeffer [6] placed †*Hemicalypterus* in the family †Semionotidae, which at the time traditionally included other hypsisomatic (deep-bodied) fishes such as †*Tetragonolepis* and †*Dapedium* [9]. Lehman [10] proposed the family †Dapediidae to unite the deep-bodied fishes †*Dapedium*, †*Tetragonolepis*, †*Dandya*, and †*Heterostrophus* (= †*Heterostropheus*), but Schaeffer [6] suggested that these lineages, as well as †*Hemicalypterus*, had independently evolved their deep-bodied appearances, and dismissed this familial union. Later authors (e.g., [11]) followed [6] and included additional deep-bodied neopterygian fishes in †Semionotidae, such as the genera †*Sargodon*, †*Dandya*, and a new species of †*Dapedium*, †*D*. *noricum*.

Other authors have maintained that the deep-bodied neopterygian fishes, such a †*Dapedium*, constitute a separate family, the †Dapediidae, first proposed by Lehman [10]. Wenz [12] and Patterson [13] also supported the separation of “dapediid-type” fishes and “semionotid-type” fishes by noting that deep-bodied fishes, such as †*Dapedium* and †*Tetragonolepis*, were so morphologically distinct from other semionotids that they do not form a natural group. Thies and Hauff [14] rediagnosed the family †Dapediidae based on shared characters, such as the deep, disc-shaped body. and considered the following genera members of †Dapediidae: †*Dapedium*, †*Paradapedium*, †*Sargodon*, †*Heterostrophus*, †*Dandya*, †*Tetragonolepis*, and †*Hemicalypterus*. However, Thies and Hauff [14] did not conduct a phylogenetic hypothesis to test the monophyly of their †Dapediidae or investigate the evolutionary relationships among taxa within the family. More recently, Thies and Waschkewitz [15], produced a phylogenetic hypothesis of evolutionary relationships of dapediids in their reexamination of †*Dapedium pholidotum*. Based on new information, they [15] could not support a sister-relationship between †*Dapedium* and teleosts proposed in other studies (e.g., [16, 17, 18]), and showed support for a relationship between dapediids and Ginglymodi. Thies and Waschkewitz [15] established a new order, †Dapediiformes, with a single family †Dapediidae, but their phylogenetic study was restricted to the genus †*Dapedium*, and failed to include other proposed dapediid taxa [14], which includes †*Hemicalypterus* [14].

Recent collecting expeditions to the Upper Triassic Chinle Formation in Lisbon Valley, San Juan County, Utah by scientists at the Natural History Museum of Utah and the St. George Dinosaur Discovery Site recovered hundreds of fish fossils, including new species as well as taxa identified by [6]. The assemblage of osteichthyan fishes from the Chinle Formation in Utah includes the palaeonisciform †*Turseodus* [6]; the redfieldiiforms †*Synorichthys*, †*Cionichthys*, and †*Lasalichthys* [6]; the enigmatic actinopterygian †*Tanaocrossus kalliokoskii* Schaeffer, 1967 [6]; recently described semionotiforms †*Lophionotus sanjuanensis* Gibson, 2013a [19] and †*Lophionotus chinleana* Gibson, 2013b [20]; and one species of coelacanth, †*Chinlea sorenseni* Schaeffer, 1967 [6]. These recent collecting efforts also recovered dozens of additional specimens of †*Hemicalypterus weiri*. These new specimens provide additional morphological and meristic data that were lacking in the original description by Schaeffer [6], and a thorough redescription of the genus is warranted to account for new, important morphological data.

The purpose of this paper is to thoroughly reexamine and redescribe †*Hemicalypterus weiri*, as well as ascertain its evolutionary relationship among neopterygians within the context of phylogenetic analyses. †*Hemicalypterus* has never been included in any phylogenetic analysis and thus its relationship to other neopterygian fishes has remained unresolved.

### Geologic Setting

Specimens of †*Hemicalypterus weiri* are found in the Upper Triassic (~210–205 million years ago [21]) Church Rock Member of the Chinle Formation in Lisbon Valley, San Juan County, southeastern Utah. The Chinle Formation in Lisbon Valley is separated into two member-level units: the Kane Springs beds and the Church Rock Member [21]. The Church Rock Member is comprised of alternating layers of mudstone, siltstone, fine-grained sandstone and conglomerate [21]. The fish-bearing beds are isolated stream deposits made of fine-grained, red and pale green sandstone layers with cross-lamination, occurring approximately 15 meters below a discontinuous, informal unit termed the ‘red ledge’ [21]. Based on geologic and lithostratigraphic studies of the Chinle Formation of southeastern Utah (e.g., [21, 22]), the Chinle Formation represents a complex freshwater fluvial-deltaic-lacustrine system with increasing aridity and a perennial monsoonal climate ([21, 23, 24]). North America at this time was climatically impacted by the breakup of Pangaea (e.g., [24]), as well as the Manicouagan bolide impact in Quebec, Canada (e.g., [25]). Both of these events potentially impacted both terrestrial and aquatic fauna of North America during the Norian Triassic [5, 26].

## Materials and Methods

Specimens of †*Hemicalypterus* from the American Museum of Natural History (AMNH), the Smithsonian National Museum of Natural History (USNM), and the Natural History Museum of Utah (UMNH) were examined for this study. Specimens from the AMNH and USNM were collected by geologists and paleontologists in the mid-20th century [6], whereas the specimens from UMNH were more recently collected between 2004–2010 by the author as well as researchers from the UMNH and the St. George Dinosaur Discovery Site (SGDS), and trained volunteers from the Utah Friends of Paleontology [8, 27]. Specimens collected for this study were collected under Utah State Institutional Trust Lands Administration permits 02–334 and 05–347 and reposited in the UMNH. In instances where the fish fossils were obscured by rock matrix, specimens were carefully prepared and exposed with the use of pneumatic tools, microjacks, and sharpened carbide needles. In a few cases when only a negative impression of the specimen was preserved, a silicone peel was made to examine a positive cast of the fossil. Silicone peels were dusted with ammonium chloride to enhance contrast. Specimens were examined using several stereomicroscopes with varying resolution power. Photographs of each specimen were taken under normal lighting conditions with a Canon digital SLR camera with macro-style lenses (65 and 100 mm). Specimens were also examined and photographed under fluorescence using a Leica stereomicroscope with GFP-LP and GFP3 filters, and a SMZ1 stereomicroscope with a P2-EFL GFP-B filter. Drawings of specimens were done with a camera lucida arm attachment and a digital drawing tablet over high-resolution photographs.

### Bone terminology

The terminology used herein follows osteological terminology outlined by Schultze [28] and Wiley [29]. Postcranial morphology is described herein using the terminology set forth by Arratia [30]. In instances where the terminology has varied between authors or over the years and does not follow the terminology outlined in the studies above, the traditional terminology will be presented in parentheses the first time that the bone is cited, to aid in interpreting homologous characters for future morphological, descriptive, and phylogenetic studies.

#### Anatomical Abbreviations

**a.pr**, ascending process of the premaxilla; **a.io**, anterior infraorbital (lacrimal); **ang**, angular; **ao**, antorbital; **ar**, articular; **bchst**, branchiostegal; **bf**, basal fulcra; **b.pr**, branched principal ray; **cl**, cleithrum; **d**, dentary; **dpt**, dermopterotic; **dsph**, dermosphenotic; **dt**, dentary teeth; **ecp**, ectopterygoid; **enp**, endopterygoid; **ep**, epural; **ex**, extrascapular; **ff**, fringing fulcra; **g**, gular; **hyp**, hypural; **io**, infraorbital; **iop**, interoperculum; **n**, nasal; **mpt**, metapterygoid; **mx**, maxilla; **op**, operculum; **p**, parietal (frontal); **pcl**, postcleithrum; **phyp**, parhypural; **pmx**, premaxilla; **pmxt**, premaxillary teeth; **pop**, preoperculum; **pp**, postparietal (parietal); **pr**, principal ray; **psph**, parasphenoid; **ptt**, posttemporal; **qu**, quadrate; **qj**, quadratojugal; **scl**, supracleithrum; **so**, supraorbital; **sop**, suboperculum; **suo**, suborbital.

#### Other Abbreviations

**HL**, head length; **MBD**, maximum body depth; **SL**, standard length.

### Morphological character and taxonomic sampling for analysis

To ascertain the phylogenetic relationships of †*Hemicalypterus weiri* to other neopterygian fishes, both parsimony and maximum likelihood cladistic analyses were performed based on a morphological character matrix of 100 characters and 54 taxa. The morphological data matrix was assembled using Mesquite Version 3.04 build 725 [31]. The data matrix is built upon [20], which used many of the characters from López-Arbarello [32] for ginglymodian fishes. Two other studies [15, 33] have also used either the whole matrix or a subsample of the matrix from [32] for their respective analyses of holostean fishes. Two additional characters (the presence of a suprapreopercular bone, and the number of infraorbitals anterior to the orbit, characters 96 and 97 in this matrix, respectively) from [33] were added to the matrix because of their use in further delineating ginglymodian relationships. Three characters include an additional character state for each, as first proposed in [15]: character 1 includes a state 4—dorsal fin originates posterior to pelvic fins and extends opposite to anal fin; character 22 includes a character state 3—length of postparietals (parietals of traditional terminology) more than half of the parietal (frontal of traditional terminology); character 23 includes a character state 2—parietals (frontals) only slightly longer than their maximum width.

Two synapomorphic characters for †Dapediidae from [14, 15] were newly included in this study: character 94—body deeply fusiform to nearly circular (absent 0, present 1); character 95—hem-like dorsal and anal fins (absent 0, present 1).

To further elucidate a relationship to teleostean fishes, three additional teleostean synapomorphic characters were added to this matrix based on [34]: character 98—tube-like, canal-bearing, anterior arm of the antorbital (absent 0, present 1); character 99—two vertebral centra fused into occipital condyle in adults (absent 0, present 1); character 100—hypural articulating with a few caudal rays (absent 0 present 1).

The following characters are newly added to this study: character 91—morphology of the premaxillary teeth (unicuspid 0, bicuspid/bifid 1, multicuspid 2); character 92—ventral ridge scales (absent 0, present 1); character 93—thickness of scales posteriad (no reduction in thickness 0, reduced in thickness or lost 1).

One character from [20] was modified to include an additional state for this study. Character 83, the morphology of the dorsal ridge scales, includes a newly defined character state 3 for scales that are toothed or spinose, meaning the present of multiple spines per dorsal ridge scale.

Coding for †*Callipurbeckia minor* was changed for character 29 from [20] and [32]: the circumorbital ring closes in ontogeny in C. minor (López-Arbarello personal communication); this character coding has been changed from 0 to 1 in this study. Coding of †*Siemensichthys* from [20] was changed for two characters based on [34]: character 7 is changed from? to 0; character 10 is changed from? to 1.

Additional holostean taxa listed below are added to the data matrix based upon examination of specimens and interpretation of previous studies; each of these taxa have been chosen because previous hypotheses have purported a potential affinity to †*Hemicalypterus*. As †*Hemicalypterus* has been hypothesized to be related to semionotids within Holostei, five additional holostean fishes were included to further resolve the relationships of holostean fishes: the ionoscopid fish †*Archaeosemionotus connectens*; ginglymodian fishes †*Lepidotes elvensis*, †*Camerichthys lunae*, and †*Kyphosichthys grandei*; and the archaeomaenid fish †*Aetheolepis mirabilis*. Scores for †*Archaeosemionotus connectens* are based on [35]; scores for †*Lepidotes elvensis* are based on [36]; scores for †*Camerichthys* are based on [33]; scores for †*Kyphosichthys* are based on [37]; scores for †*Aetheolepis mirabilis* are based on [38] and examination of photographs of specimens.

Other studies as outlined above have hypothesized a close relationship between †*Hemicalypterus* and dapediid fishes, and so several taxa are included in the analysis that are putative members of †Dapediidae (†Dapediiformes): †*Sargodon tomicus*, †*Dapedium caelatum*, †*Dapedium stollorum*, †*Dapedium punctatum*, †*Dapedium pholidotum*, †*Dandya ovalis*, †*Paradapedium egertoni*, †*Tetragonolepis oldhami*, †*Tetragonolepis semicincta*, and †*Heterostrophus phillipsi*. Character scores of each taxon for the morphological data matrix are based on the following: scores for †*Tetragonolepis semicincta* are based on [39] and personal examination of specimens; scores for †*Tetragonolepis oldhami* and †*Paradapedium egertoni* are based on [40]; scores for †*Sargodon tomicus* and †*Dandya ovalis* are based on [11]; scores for †*Dapedium stollorum* and †*Dapedium punctatum* are based on [14, 15, 20, 41] and personal examination of specimens; scores for †*Dapedium pholidotum* are based on [15] and personal examination of specimens; scores for †*Heterostrophus phillipsi* are based on [42, 43] and examination of high-resolution photographs of the type material, freely provided by the JISC GB3D Type Fossils Online Project (found at http://www.3d-fossils.ac.uk).

Please see the Electronic Supplemental Information documents for the complete list and description of phylogenetic characters and specimen materials examined (S1 Text) and morphological data matrix (S1 Table).

### Phylogenetic analysis of morphological data

A parsimony-based analysis was performed using PAUP* Version 4.0a147 for Macintosh [44]. Characters were unordered and given equal weight. Multistate characters were treated as polymorphisms. The starting tree in the heuristic search was obtained via stepwise addition, and branch-swapping was done with tree-bisection-reconnection (TBR). Branches were collapsed if maximum branch length was zero. Symmetric resampling bootstraps [45] were performed with 100 replicates. The subholostean †*Perleidus* was designated as the outgroup taxon in the analyses.

A maximum likelihood analysis was conducted using the software Garli Version 2.01 [46] using a single partition with the Lewis MK model modified for only variable characters (MKv) as recommended for morphological data [47]. Characters were unordered, and polymorphisms were treated as missing data. Five separate likelihood analyses were conducted, with the tree having the best likelihood score presented here in order to evaluate evolutionary relationships. A nonparametric bootstrap analysis [45] was performed on the dataset with 100 random pseudoreplicates. As in the parsimony analysis, †*Perleidus* was rooted as the outgroup.

## Results

### Systematic Paleontology

Osteichthyes Huxley, 1880 [48]

Actinopterygii Cope, 1871 [49]

Neopterygii Regan, 1923 [50]

Holostei Müller, 1845 [51] (*sensu* Grande, 2010 [52])

†Dapediiformes Thies and Waschkewitz, 2015 [15]

†Dapediidae Lehman 1966 [10] (*sensu* Thies and Hauff, 2011 [14])

Genus †*Hemicalypterus* Schaeffer, 1967 [6]

#### Generic etymology

Greek origin, meaning *hemi* for “half” and *kalyptos* for “covered”, referring to the conspicuous body scales covering the anterior portion of body only.

#### Generic Diagnosis

Same as for type and only species.

#### Type species

†*Hemicalypterus weiri* Schaeffer, 1967 [6] from the Upper Triassic Chinle Formation of San Juan County, Utah (United States)

†*Hemicalypterus weiri* Schaeffer, 1967

Figs 1–8

Schaeffer [6]: Fig 12; pls. 24–25

**Fig 1:**
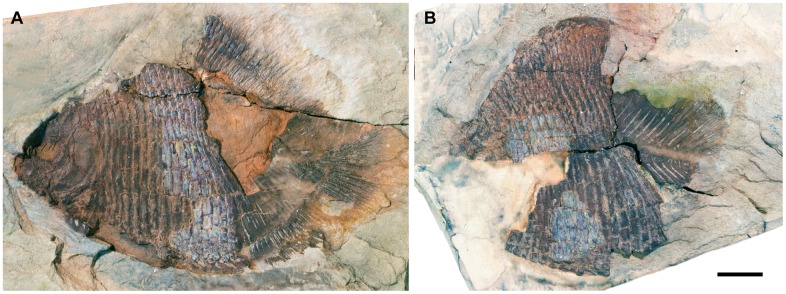
†*Hemicalypterus weiri* holotype specimen, USNM V 23427. Part and counterpart each in left lateral view. (A) USNM V 23427A. (B) USNM V 23427B (image reversed). Scale bar equals 1 cm.

**Fig 2:**
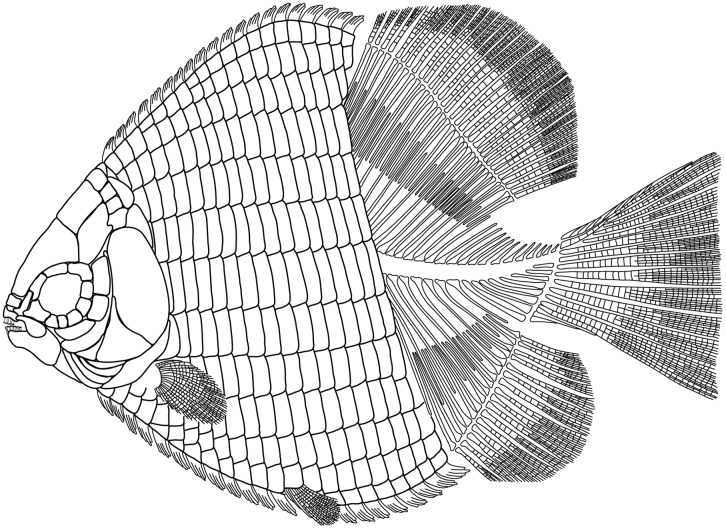
Reconstruction of †*Hemicalypterus weiri*. Modified from Schaeffer [6] and Gibson [5].

**Fig 3:**
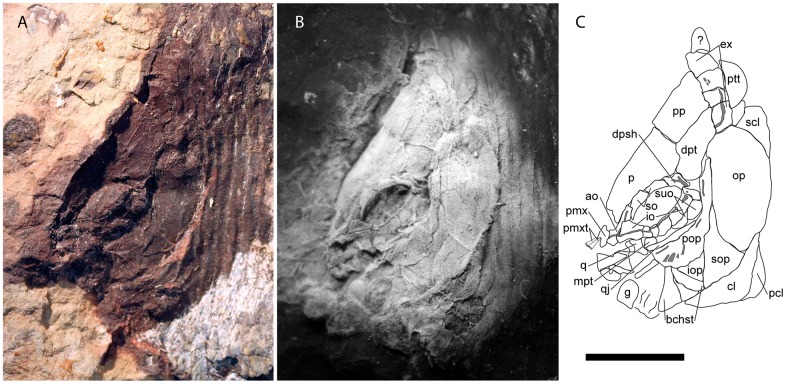
Skull of †*Hemicalypterus weiri*, specimen AMNH 5718A. (A) Photograph of the skull of specimen AMNH 5718A under normal lighting in left lateral view. (B) Photograph of a silicone peel of AMNH 5718A, dusted with ammonium chloride to provide contrast. (C) Drawing interpretation of AMNH 5718A with bones of the skull labeled. Scale bar equals 1 cm.

Gibson [5]: Figs 1–3

#### Specific etymology

Named after Gordon W. Weir, a geologist for the United States Geological Survey who surveyed the geology of San Juan County, and was one of the first to report the presence of fossil fishes in Lisbon Valley, San Juan County.

#### Holotype

USNM V 23425A, B: nearly complete specimen in lateral aspect (Fig 1)

#### Referred specimens

AMNH 5709A, B: nearly complete specimen in lateral aspect; AMNH 5710: three partial specimens; AMNH 5711: anterior half of specimen; AMNH 5712: anterior half of specimen; AMNH 5713: partial specimen, head dissociated; AMNH 5714: partial trunk; AMNH 5715: partially dissociated trunk; AMNH 5716: nearly complete specimen; AMNH 5717: trunk and dissociated skull; AMNH 5718: anterior portion of specimen; MCZ 9034: partial specimen; UMNH VP 19419: nearly complete specimen, lacking caudal fin, on block with †*Lophionotus sanjuanensis*; UMNH VP 22903: partial specimen, lacking posterior half; UMNH VP 22904: nearly complete specimen, lacking caudal fin; USNM V 23422: specimen lacking posterior portion; USNM V 23423: skull and dorsal region of trunk; USNM V 23424: partial articulated body, lacking anterior portion of skull; USNM V 23426: partly dissociated specimen; USNM V 23427: anterior portion of fish, nearly complete skull; USNM 23428: nearly complete specimen, lacking caudal fin; USNM V 23429, nearly complete specimen, lacking caudal fin.

#### Specific diagnosis

Amended from [6] to account for additional diagnostic information. This species is based upon the unique combination of the following characters: small (average 65 mm SL), hypsisomatic (deep-bodied) fish, nearly cycloidal, laterally compressed body shape; anterior half of body covered in ganoid scales; posterior half of body completely scaleless; no central ossification around notochord; prominent dorsal and ventral ridge scales along anterior half of body; each dorsal and ventral ridge scale possesses strongly denticulated distal margins; premaxillary and dentary teeth multicuspid, with long cylindrical bases and laterally compressed crowns with 3–5 cusplets per tooth; maxilla edentulous with small anterior arm and broader, rounded posterior margin; maxilla not extending beyond middle of orbit; vertically-oriented preoperculum with broad, paddle-like ventral process; lateral origin of pectoral fins; dorsal and anal fins lacking basal or fringing fulcra, hem-like.

### Description

†*Hemicalypterus weiri* is a small-sized ganoid fish, with a deep, laterally compressed, cycloidal body shape (Figs 1 and 2). Average length of specimens examined is approximately 65 mm SL, with the smallest specimen obtaining a SL of 53 mm and the largest with a SL of 85 mm. The TL of complete specimens averages 78.4 mm. The highest point on the dorsal margin occurs anterior to the dorsal fin, and the lowest point on the ventral margin occurs just posterior to the placement of the pelvic fins. The MBD, measured from the lowest point of the body vertically, averages at 57.25 mm. The anterior portion of the flank is covered in rectangular ganoid scales, which terminate just anterior to the anterior margins of the dorsal and anal fins (Figs 1 and 2). The edge of the squamation terminates in an oblique angle anterodorsally to posteroventrally. The posterior portion of the trunk and the caudal peduncle are scaleless, and the axial skeleton is exposed.

#### Skull roof

**Fig 4:**
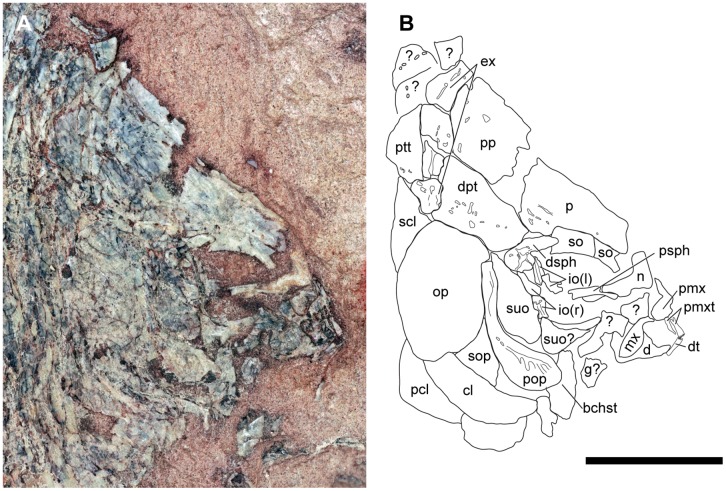
Skull of †*Hemicalypterus weiri*, specimen USNM V 23427. (A) Photograph of the skull of USNM V 23427 in right lateral view. (B) Drawing interpretation of USNM V 23427 with bones of the skull labeled. Scale bar equals 1 cm.

The skull roof contains a pair of parietals (frontals) and postparietals (parietals). The parietals comprise the bulk of the skull roof, and are semi-triangular in shape with a concave embayment along the lateral margin where it articulates with the supraorbitals (Figs 2–4). The anterior margin is weakly digitate. The median suture between the parietals is linear, and the parietals articulate posteriorly with the postparietals in a digitate suture and posterolaterally with the dermopterotics (Figs 3 and 4).

The postparietals are rectangular, slightly longer than wide, and are about half of the length of the parietals. The surface of the postparietals is ornamented with weak crenulations. They articulate with the parietals anteriorly in a digitate suture, with the dermopterotics laterally, and with the median extrascapulars posteriorly (Figs 2–4).

The supraorbital canal passes along the lateral margin of the parietal and into the postparietal. On the parietal, the canal follows the lateral margin of the bone and passes downward on the posterior expansion, but does not appear to connect to the canal present in the dermosphenotic (Figs 3 and 4).

The dermopterotics are situated laterally to the postparietals on the skull roof (Fig 2). Each dermopterotic is nearly equal in size, if not bigger than the postparietals. Each dermopterotic is longer than deep and is rhomboid in shape, with the medial and lateral margins being longer than the anterior and posterior margins. The medial margin articulates with the posterolateral margin of the parietal and the lateral margin of the postparietal, the anterior margin articulates with the posterior margin of the dermosphenotic. Laterally, each dermopterotic articulates with the dorsal part of the preoperculum and operculum. Posteriorly, the dermopterotic has a rounded edge with a small notch in the ventral corner, and appears to overlap the lateral extrascapular. The dermopterotic bears the temporal canal running from the dermosphenotic into the extrascapulars (Figs 3 and 4).

The extrascapulars are only partially preserved in the type specimen USNM V 23425, but are well preserved in USNM V 23427 and AMNH 5718B (Figs 3 and 4). They occur in four pairs (eight bones across the skull in total). The size and shape of each extrascapular varies between the available specimens, but are all roughly quadrangular (Fig 2). The ventrolateral extrascapular articulates with the dorsal margin of the operculum and the posterior margin of the dermopterotic, and carries the temporal canal from the dermopterotic, which curves dorsad and traverses through the remainder of the extrascapular series. In USNM V 23427, the sensory canal branches in the lateralmost extrascapular, with one branch, the supraoccipital commissure, traversing dorsad/mediad through the extrascapular series, and the other branch traversing posteriad to connect with the main lateral line along the posttemporal (Fig 4).

Posterior to the extrascapulars, one pair of posttemporals lies along the skull roof (Fig 2). In AMNH 5718A (Fig 3), USNM V 23427 (Fig 4) and USNM V 23428, the posttemporals are long and semi-triangular, tapering mediad towards the skull roofs median, and broadening laterad. The posterior margin of the posttemporals is curved, and its ventral edge is concave and articulates with the dorsal convex surface of the supracleithrum. In USNM V 23427, the lateral line canal passes through the lateral half of the posttemporal.

#### Snout and Cheek Region

**Fig 5:**
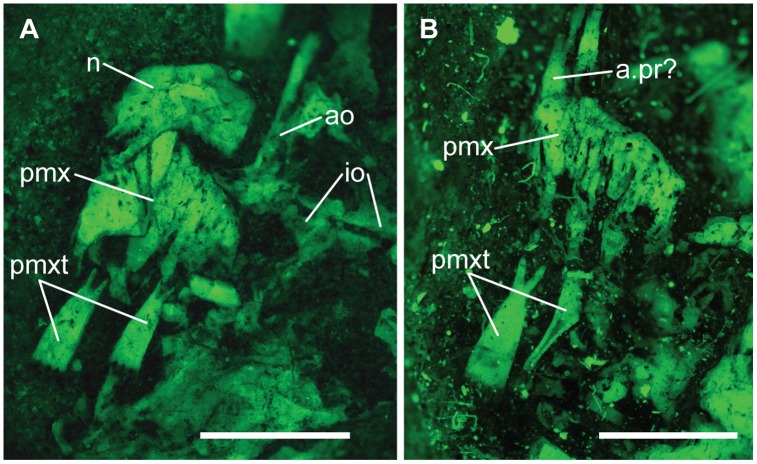
Premaxilla and snout of †*Hemicalypterus*. (A) AMNH 5712A, photographed under fluorescence. (B) USNM V 23427, photographed under fluorescence. A possible ascending process of the premaxilla is indicated with **a.pr**. Scale bars equal 1mm.

†*Hemicalypterus weiri* has a complete (closed) circumorbital ring. The type specimen (USNM 23425A, Fig 1) appears to have a series of three to four infraorbitals posterior and ventral to the orbit, but AMNH 5718A clearly has at least six infraorbitals (Fig 3). Beginning at the ventral margin of the dermosphenotic, there are at least one or two small quadrangular infraorbitals posterior to the orbit and anterior to the suborbitals (Fig 3). The two infraorbitals posteroventral to the orbit are deeper than those posterior to the orbit, and then the series narrows anteriad to a series of small infraorbitals (number varies from two to three) directly below the orbit before it contacts the large, trapezoidal anteriormost infraorbital (lacrimal/lachrymal). The infraorbital series bears the infraorbital canal near the orbital margin of each bone, although the canal becomes more medial in the anteriormost infraorbital into the antorbital bone. The anteriormost infraorbital (lacrimal/lachrymal) is larger than the rest of the infraorbital bones in the series, and expands to occupy the anteroventral corner of the orbit and contact the anterior supraorbital (Fig 3).

The snout bones of †*Hemicalypterus* include paired nasals and antorbitals. One of the nasal bones is well-preserved in AMNH 5712A (Fig 5), and it lies anterior to the parietals, though it does not appear to directly articulate with the parietals or the premaxillae; rather it is situated above the premaxilla (Fig 5). The nasal bone is rectangular on the lateral edge. The antorbital bone is visible in lateral view on AMNH 5712A and 5718A (Figs 3 and 5). It articulates posteriorly with the anteriormost infraorbital (Fig 5) and has a long, straight dorsal process that carries the supraorbital canal. There is a short, tubular process at the anteroventral edge of the bone that is directed anteromediad (Figs 3 and 5). This process does not appear to be as elongate as the tubular antorbital process seen in semionotiforms. The median rostral bone is not visible on any specimen.

The dermosphenotic is located at the posterodorsal corner of the orbit, where it meets the infraorbital series on the ventral edge, and the supraorbitals anteriorly (Figs 3 and 4). Dorsally it articulates with the parietal and posterodorsally with the dermopterotic. The dermosphenotic is small and semi-triangular in shape and bears the junction of the infraorbital, supraorbital, and temporal canals. Anterior to the dermosphenotic are two elongated, rectangular supraorbitals (Figs 3 and 4). On AMNH 5718A, the posterior supraorbital has a short row of tubercles on the orbital edge. The anterior supraorbital articulates with the anteriormost infraorbital, completing the circumorbital ring. Both supraorbitals articulate along their dorsal margins with the lateral edge of the parietal, and together are shaped slightly convex, curving around the orbit (Figs 3 and 4).

Three anamestic suborbitals occupy the space between the infraorbitals and the anterior margin of the preoperculum in the holotype USNM V 23425 (Figs 1 and 2), and there are at least two suborbitals preserved in USNM V 23427 (Fig 4) and AMNH 5718A (Fig 3). The dorsal suborbital posterior to the infraorbitals and anterior to the dorsal process of the preoperculum is large and quadrangular in USNM V 23427 (Fig 4), USNM V 23428, and AMNH 5716, with an elongated, tapered dorsal margin (Fig 4). In other specimens, such as holotype USNM V 23425 and AMNH 5718A, the dorsal suborbital is smaller, but retains a similar triangular shape (Figs 1 and 3). In the holotype USNM V 23425 (Fig 1) and USNM V 23422, there are two more suborbitals in the series anteroventral to the suborbital described above, each one quadrangular and occupying the space between the preoperculum and the infraorbitals; in other specimens (e.g., UNSM V 23427, AMNH 5718), there is only one suborbital preserved ventral to the dorsal suborbital (Figs 3 and 4). In these specimens the ventral suborbital is small and triangular in shape.

The preoperculum is a vertical bone, composed of a narrow tall dorsal process and a broad, flattened, paddle-like anteroventral process (Figs 1, 3 and 4). It is bordered posteriorly by the operculum, ventrally by the suboperculum and interoperculum, anterodorsally by the dermopterotic and the suborbital series. The preopercular sensory canal is preserved as a groove that runs near the anterior margin of the bone, with the ventral process perforated by ventrally directed grooves, which house exits for branches of the preopercular sensory canal. The preopercular sensory canal exits anteriad from the ventral process of preoperculum.

The operculum is large and oval, shorter than deep. Its surface is covered with radiating ridges originating around the upper middle portion of the bone. It articulates dorsally with the dermopterotic, extrascapular and supracleithrum, anteriorly with the preoperculum, anteroventrally with the suboperculum, and posteroventrally overlies the cleithrum (Figs 1, 3 and 4).

The suboperculum is triangular, and is overlain by the operculum; the exposed depth of the suboperculum is less than half the depth of the operculum. It has a narrow ascending process that inserts into the space between the preoperculum and operculum. The anterior margin articulates with preoperculum and interoperculum (Figs 1, 3 and 4).

The interoperculum is a small, triangular bone situated between the preoperculum and suboperculum. Its anterior tip does not reach the lower jaw articulation, but it is separated from the jaw by lower process of the preoperculum (Fig 3).

#### Jaws

The premaxillae are small and may lack ascending processes that would otherwise articulate with the parietals. However, the premaxilla on USNM V 23427 appears to have a small ascending process at the anteromedial margin of the bone (Figs 4 and 5). Each premaxilla bears two to three individual teeth with long cylindrical bases, and spatulate crowns with multidenticulate edges. These broadened edges contact their neighbors to create a continuous cutting edge (Figs 3–5). These teeth are described in greater detail in [5] and not described further here.

As observed in lateral view in USNM V 23427, the maxilla is a wedge-shaped bone with a curved, saddle-shaped ventral margin (Fig 4). It tapers anteriorly to a small, rounded process. The posterior two-thirds of the bone is broad and laterally flattened. The caudal margin is curved with a slight taper caudad. The maxilla is edentulous and appears to lack ornamentation on the dermal surface. In USNM V 23427 the maxilla is preserved in its “extended” position (the posterior portion swung forward pivoting from the anterior process), and is laying on top of the lower jaw. In “resting” position (the posterior process more horizontal), the length of the maxilla would only extend slightly below the orbital region, and not reach the midline of the orbit.

The lower jaw is short and robust. Like the teeth of the premaxilla, the teeth of the lower jaw are multidenticulate, and number at least six. Each tooth has a long cylindrical base that extends deep into the jaw (visible through fluorescent images; [5]: Fig 2), and broadens at the crown to a spatulate margin with a multicuspid edge with four individual, styliform cusps. Each tooth contacts its neighbor, and each tooth is slightly recurved, making a continuous, scraping scoop. The teeth reduce in size posteriad. The coronoid process is large on UMNH VP 19419, but the posterior portion of the lower jaw is poorly preserved on all of the specimens. The mandibular canal can be viewed on UMNH VP 19419 as a series of pores passing along the ventral margin of the bone ([5]: Fig 2).

A single gular plate is preserved on AMNH 5718A (Fig 3) and USNM V 23427 (Fig 4). It is disarticulated in both specimens, but is quadrangular in shape.

The quadratojugal is clearly visible on AMNH 5718A (Fig 3). It is long and splint-like, and runs along the dorsal edge of the ventral process of the preoperculum. The rounded anterior tip of the quadratojugal articulates with the quadrate. The quadrate is triangular, with a rounded, concave posteroventral edge that cups the quadratojugal articulation (Fig 3).

A few palatal elements may also be visible on AMNH 5718A (Fig 3). The metapterygoid is visible where the suborbitals are missing ventral to the orbit, but it’s exact shape is undeterminable. It lies dorsal to the quadratojugal. Likewise, a small portion of the entopterygoid is visible between the quadrate and the metapterygoid, dorsal to the quadratojugal, but preservation prevents further examination.

The hyoid arch is not visible in any specimens.

The branchiostegals are preserved as a series of plates ventral to the interoperculum and suboperculum. Schaeffer [6] listed four branchiostegals present, but only three are observed on the holotype (Fig 1), and two on AMNH 5718 (Fig 3). They are elongate and triangular and there are anywhere from three to four preserved on the available specimens.

#### Postcranial skeleton

The cleithrum is a large, long, crescent-shaped bone, widest at the center and tapering gradually to the ends. It is partially obscured dorsally by the operculum (Fig 4). A prominent ridge runs parallel to the length of the bone (AMNH 5718A; Fig 3). A triangular supracleithrum is visible posterodorsal to and partially obscured by the operculum (Figs 3 and 4). A postcleithrum is present behind the cleithrum and is visible in the holotype USNM V 23425 (Fig 1), USNM V 23427 (Fig 4), and AMNH 5718A (Fig 3).

**Fig 6:**
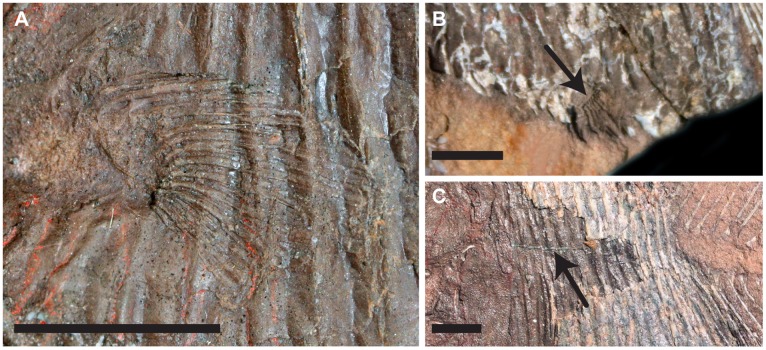
Paired fins and lateral line of †*Hemicalypterus*. (A) USNM V 23428, closeup of pectoral fin, in left lateral view. (B) USNM V 23429, poorly preserved pelvic fin as indicated by black arrow, left lateral view. (C) AMNH 5709A, posterior portion of skull and some of the flank in left lateral view (image reversed), lateral line indicated by black arrow. Scale bars equal 5 mm.

A pectoral fin is visible on AMNH 5709A, and visibly well-preserved on USNM V 23428A (Fig 6A). The position of the fin is low on the lateral side of the flank. On USNM V 23428A, two basal fulcra appear to be present on the anterodorsal edge of the pectoral fin as it is preserved (Fig 6A). There are 16 fin rays preserved, each ray bifurcating distally.

The pelvic fins are not preserved on many specimens, only a small portion of them are poorly preserved on the holotype USNM V 23425 (Fig 1) and USNM V 23429B (Fig 6B). They are located along the ventral margin of †*Hemicalypterus*, inserting midway between the skull and the origin of the anal fin, approximately 10–11 scale rows posterior to the skull and approximately eight rows anterior to the end of the squamation. Each pelvic fin consists of approximately eight fin rays, and it is unclear if the pelvic fins possess basal or fringing fulcra due to the incomplete preservation of the available specimens.

The dorsal and anal fins originate just posterior to the termination of the squamation on the flank of †*Hemicalypterus* (Fig 1). Both fins lack basal and fringing fulcra. The dorsal fin begins with short lepidotrichia, which gradually become longer posteriad until the center of the fin, and then gradually taper, creating a curved convex silhouette in lateral view. The dorsal fin has approximately 27–29 segmented, birfurcated rays preserved in AMNH 5713A, one of the larger specimens of †*Hemicalypterus*. The holotype USNM V 23425 possesses 32 visible dorsal fin rays (Fig 1). The anal fin bears resemblance to the dorsal fin, but is smaller and possesses fewer rays, having only 16 segmented, bifurcated rays in AMNH 5713A. The pterygiophores are visible in specimens of †*Hemicalypterus*, and articulate one to one with the lepidotrichia along the body margin. The number of lepidotrichia and pterygiophores are double the number of neural and haemal spines, and so the pterygiophores are interspersed in groups of two between each successive spine, and rest along the anterior and posterior sides of each spine for added fin support.

**Fig 7:**
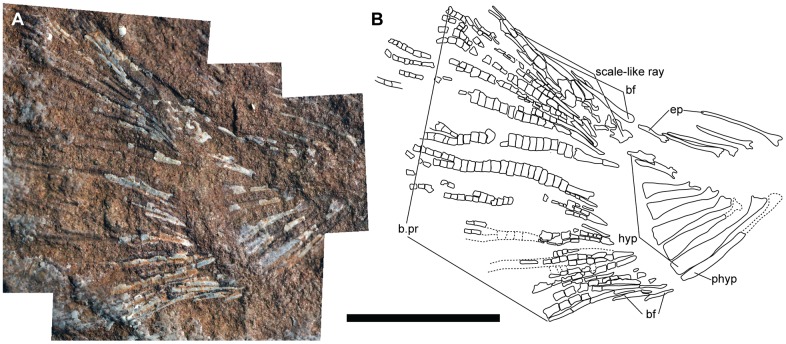
Caudal skeleton of †*Hemicalypterus*. (A) Specimen AMNH 5709A, photographed under normal lighting in right lateral view. (B) Drawing interpretation of AMNH 5709, a combination of AMNH 5709A and B to account for material on both part and counterpart. Scale bar equals 1 cm.

The caudal fin is well preserved on the holotype USNM V 23425 (Fig 1) as well as AMNH 5709 (Fig 7). The series of hypurals are arranged in a hemiheterocercal shape and the fin possesses approximately 14–16 segmented, bifurcated rays. The dorsal margin of the caudal fin possesses a row of fringing fulcra along a single, specialized scale-like ray (Fig 7), and the ventral margin of the caudal fin is bordered by two to three strong basal fulcra (Fig 7).

The scales of †*Hemicalypterus* are rectangular to rhomboidal in shape and cover only the anterior portion of the flank, ending abruptly anterior to the origin of the dorsal and anal fins (Fig 1). There are approximately 18–19 rows of scales. Scales are deepest (at least 2.5–3 times deeper than their length) at the central portion of the flank, near the lateral line scale, and scales become more shallow towards the dorsal and ventral margins. The surface of each scale is covered with slight crenulations (AMNH 5709B) or small pits (AMNH 5718B). The lateral line passes from the posttemporal and postcleithrum along the mid-flank scales (near the midline), and is visible as a prominent groove along the surface of the scales (AMNH 5709A, Fig 6C).

**Fig 8:**
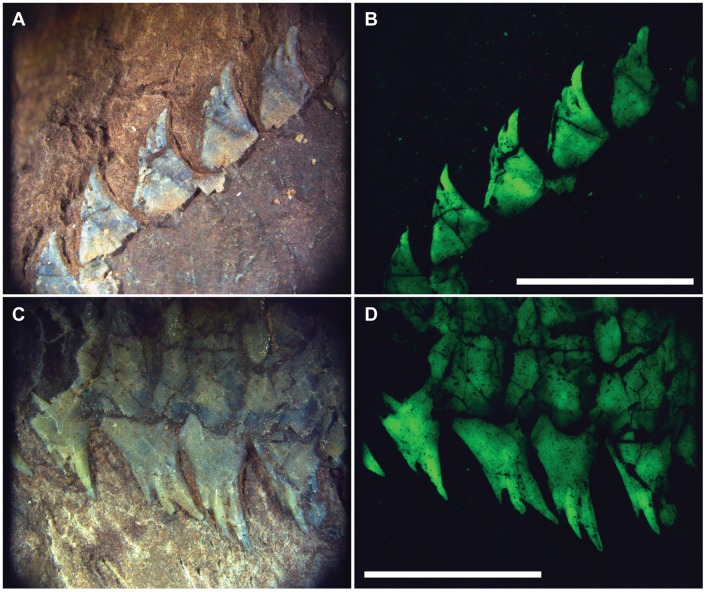
Pronounced dorsal and ventral ridge scales of †*Hemicalypterus*. The pronounced dorsal and ventral ridge scales of †*Hemicalypterus*, showing multiple, toothlike projections on each scale. Specimen UMNH VP 19419 dorsal ridge scales photographed under (A) normal lighting and (B) fluorescence. Specimen UMNH VP 19419 ventral ridge scales photographed under (C) normal lighting and (D) fluorescence. Scale bars equal 5 mm.

†*Hemicalypterus* possesses very prominent dorsal and ventral ridge scales. Each scale possesses 3–4 toothlike, spinose projections which point posteriad. These projections stand more “upright” near the skull, and become more flush with the body margin posteriad (Fig 8).

The endoskeleton is exposed on the posterior portion of the flank, where the squamation terminates (Fig 1). This exposed portion indicates that *Hemicalypterus* did not have ossified centra, but instead possessed an unrestricted notochord that supported neural arches dorsally and haemal arches ventrally. The neural and haemal arches have broad margins that would have contacted the notochord, are triangular in shape with anterior and posterior processes that contact each respective neighboring arch, and are fused to long, thin neural and haemal spines, respectively. Each spine tapers to a point, and becomes shorter posteriad. Any possible supraneurals and ribs are obscured by the squamation of the anterior portion of the flank.

The endoskeleton of the caudal fin is visible on the holotype USNM V 23425 (Fig 1), AMNH 5709 (Fig 7), AMNH 5713, AMNH 5716, and partially on UMNH VP 19419. The distinction between hypural and preural haemal spines is difficult to make on laterally compressed fossils, and the preservation is incomplete on some specimens, making it difficult to interpret the area where bifurcation occurs. However, it is estimated that *Hemicalypterus* possesses at least three to four “epurals” and seven hypurals (Fig 7). Each hypural, rather than tapering to a fine point, broadens distally to a flattened edge. The hypurals decrease in size as they curve upwards with the shape of the notochord (Fig 7). The hypural spines articulate and support the rays of the caudal fin in a one-to-one relationship, with the exception of the ventralmost hypurals and parhypural, which appear to each articulate with two caudal fin rays. This is similar to what is observed in †*Semionotus elegans* as described in [53].

### Evolutionary History of †Dapediiformes

**Fig 9:**
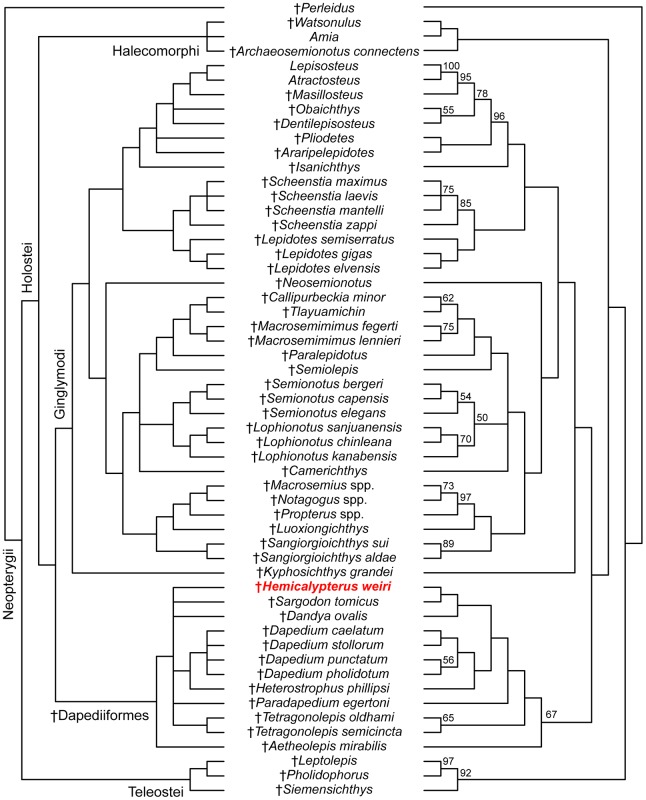
Strict consensus (left) and 50% majority rule (right) of 84 most parsimonious trees (100 characters, 55 taxa). Tree length = 417; consistency index (CI) = 0.345; retention index (RI) = 0.706; homoplasy index (HI) = 0.679. Bootstrap values >50% are given above each respective node on the majority rule tree. The position of †*Hemicalypterus* is indicated in red.

**Fig 10:**
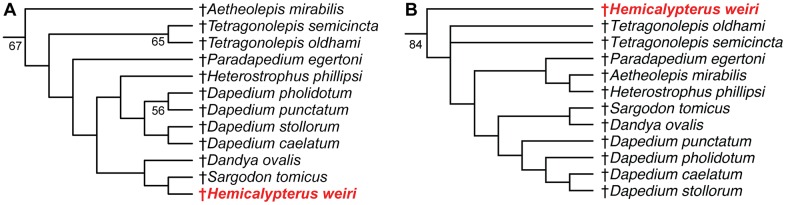
Comparison of the results of parsimony and maximum likelihood analyses. (A) The clade †Dapediiformes from the 50% majority rule parsimony tree in Fig 9; (B) the clade †Dapediiformes from the tree result obtained using maximum likelihood. Numbers at nodes indicate bootstrap support >50%. The position of †*Hemicalypterus* is indicated in red.

The parsimony analysis inferred 84 most parsimonious trees with 417 steps. The strict consensus tree and 50% majority rule consensus trees are presented in Fig 9. In the parsimony analysis, the order †Dapediiformes is recovered as a monophyletic group with a bootstrap support of 67, and sister to Ginglymodi (Fig 9) [20, 32, 52]. Ginglymodi + †Dapediiformes is sister to Halecomorphi, forming the Holostei, which is then sister to Teleostei (Fig 9). †*Hemicalypterus* is consistently recovered within †Dapediiformes in PAUP* in both strict consensus and majority rule phylogenetic trees (Fig 9). However, many relationships are unresolved within †Dapediiformes in the strict consensus. The 50% majority-rule consensus tree provides resolution of evolutionary relationships within †Dapediiformes, and infers †*Hemicalypterus* as the sister group to †*Sargodon*, and forming a clade with †*Dandya* (Fig 9). In both strict consensus and majority rule trees, the genus †*Dapedium* is monophyletic and sister to †*Heterostrophus*. Two species of †*Tetragonolepis* are recovered as monophyletic with a bootstrap support of 65 (Figs 9 and 10A).

The results of the maximum likelihood analysis conducted in Garli also recovered †*Hemicalypterus* within a monophyletic †Dapediiformes supported by a high bootstrap support of 84 (Fig 10), with †Dapediiformes sister to Ginglymodi, consistent with the results of the parsimony analysis. Relationships within †Dapediiformes differed in maximum likelihood when compared to parsimony, and interrelationships within †Dapediiformes have low bootstrap support. Fig 10 compares the differences within †Dapediiformes between the 50% majority rule consensus tree (Fig 10A) and the maximum likelihood tree (Fig 10B). Within the monophyletic genus †*Dapedium*, †*D*. *stollorum* and †*D*. *caelatum* are still recovered as sister taxa, but †*D*. *pholidotum* and †*D*. *punctatum* are pectinate. †*Sargodon* and †*Dandya*, which were recovered sister to †*Hemicalypterus* in the parsimony analysis, retain a close affinity to each other and to †*Dapedium*. †*Heterostrophus* and †*Aetheolepis* are recovered a sister taxa to †*Paradapedium*. The two species of †*Tetragonolepis*, which were recovered as monophyletic in the parsimony analysis with moderate bootstrap support (BSS = 65), were unresolved in the maximum likelihood analysis. Notably, †*Hemicalypterus* is recovered as a stem taxon within †Dapediiformes in maximum likelihood, whereas in parsimony it was recovered higher in a clade including †*Dapedium*, †*Heterostrophus*, †*Sargodon*, and †*Dandya*.

## Discussion

### Taxonomic comparisons and remarks

†*Hemicalypterus* possesses one unique character unknown in any other lower actinopterygian fish. As Gibson [5] notes, the novel tooth and jaw morphology in †*Hemicalypterus* (Fig 5) is similar to many extant fishes that feed predominantly on algae, using specialized multidenticulate teeth to scrape algae or attached organisms off of a rocky substrate. Gibson [5] gave several extant examples in both marine (e.g., *Acanthurus*, *Zebrasoma*, *Siganus*) and freshwater (e.g., *Labeotropheus*, *Maylandia*, *Hyphressobrycon*) communities that possess specialized multicuspid dentition similar to what is observed in †*Hemicalypterus*. In the fossil record, this type of tooth morphology is found in teleosts in the Eocene Monte Bolca Formation [4, 54], and in some isolated remains of fishes of unknown actinopterygian affinity in Cretaceous deposits from Asia (e.g., [55]) and northern Africa (e.g., [56, 57]). To date, †*Hemicalypterus* is the oldest known ray-finned fish with this specialized multicuspid dentition, and demonstrates that ray-finned fishes were diversifying into and exploiting novel ecological niches (e.g., herbivory or partial herbivory) far earlier than previously hypothesized by other studies (e.g., [5, 54]).

Visually, †*Hemicalypterus* is most similar to †*Aetheolepis mirabilis*, a deep-bodied fish from the Jurassic Talbragar beds of Australia [38], which was placed in the family †Semionotidae by Woodward [38], but later moved to the family †Archaeomaenidae by Gardiner [16]. Like †*Hemicalypterus*, †*Aetheolepis* possesses thick rhombic ganoid scales on the anterior portion of the flank and a distinct reduction/loss of scales on the posterior portion of the flank. However, †*Aetheolepis* displays thin amioid-type scales on the posterior flank, whereas †*Hemicalypterus* is scaleless on the posterior portion of the flank (Figs 1 and 2). The dorsal and anal fins of †*Aetheolepis* are triangular, with the longest rays at the anterior portion of the fin and reducing in length posteriad, whereas the fin rays of †*Hemicalypterus* begin short at the origin, reach their greatest length near the center, and then shorten slightly posteriad (Figs 1 and 2). The distal margins of the dorsal and anal fins are rounded. †*Aetheolepis* lacks the prominent spinose dorsal and ventral ridge scales seen on †*Hemicalypterus* (Fig 8), and the caudal fin of †*Aetheolepis* lacks the dorsal upturn of the notochord and hypurals as seen in †*Hemicalypterus* (Fig 7); rather the notochord remains centered as it enters the caudal region. Detailed comparison of the skull features of †*Aetheolepis* and †*Hemicalypterus* is not possible at the time of this study.

†*Hemicalypterus* has been placed in †Dapediidae (†Dapediiformes) in previous studies (e.g., [14]), and shares with dapediid fishes (such as †*Dapedium*, [14, 15]): a deep, disc-shaped body (character 94); hem-like dorsal and anal fins (character 95); presence of prominent dorsal (character 83) and ventral ridge scales (character 92); and dorsal fins with more than 20 rays (character 77). †*Hemicalypterus* possesses some features not seen in †*Dapedium*, such as the prominence of the interoperculum; in †*Dapedium* the interoperculum is large and spans the length of the ventral arm of the preoperculum, reaching the jaw symphysis; in †*Hemicalypterus*, the interoperculum is small and isolated from the jaw symphysis (Figs 2 and 3). The dorsal arm of the preoperculum is a much more prominent part of the cheek region of †*Hemicalypterus* (Figs 1–4) than in †*Dapedium*, where the dorsal arm of the preoperculum is almost entirely covered by suborbital bones in all species. †*Hemicalypterus* is also very distinct from †*Dapedium* with regard to squamation: †*Dapedium* retains thick ganoid scales across the entire flank, as opposed to the scaleless posterior half of †*Hemicalypterus* (character 93). With regard to dentition, some species of †*Dapedium* display bifid marginal teeth on the premaxilla (e.g., †*D*. *punctatum*), but the toothlets of bifid teeth of some dapediids are much more robust than the fine, multicuspid toothlets of †*Hemicalypterus* (Figs 3–5), and were possibly used for durophagous feeding by plucking invertebrates from substrate, rather than for herbivory. The fine toothlets of †*Hemicalypterus* suggest weak benthic feeding, and would have likely broken off if used for durophagy [5]. There is also no evidence of crushing palatal teeth in †*Hemicalypterus*. Other species of †*Dapedium* (e.g., †*D*. *stollorum*) possess unicuspid, styliform teeth likely used for generalist feeding strategies [14].

†*Sargodon tomicus* [11] from the Upper Triassic of Lombardy, Italy is another species of deep-bodied dapediid fish that possesses many similarities to †*Hemicalypterus*. †*Sargodon* possesses a spinose ventral ridge scales, however the dorsal ridge scales lack large spines. There is a reduction in the thickness of scales toward the caudal peduncle, but †*Sargodon* retains rhombic scales along the entire flank; scales on the ventral portion of the body are dorsoventrally longer than those on the dorsal half of the flank (above the lateral line). The general body shape is deep, but more diamond-shaped than the rounded body of †*Hemicalypterus* (Figs 1 and 2). The dorsal and anal fins are elongated, but the fin rays are longest at the anterior origins and become shorter posteriad. There are many differences in the head, for example the preoperculum of †*Sargodon* is not exposed on the dermal surface, whereas it is nearly fully exposed and is a large component of the cheek region of the head of †*Hemicalypterus*. The dentition of †*Sargodon*, like some dapediids, is comprised of bifid incisors on the premaxilla and dentary, as well as palatal grinding teeth, indicating that it fed on durophagous invertebrates, possibly using the bifid marginal teeth to pluck invertebrates from a substrate, then crushing hard exoskeletons with the grinding palatal teeth. This is unlike †*Hemicalypterus*, which possesses only multicuspid teeth on the premaxilla and dentary, and shows no signs of a grinding palatal dentition.

### Phylogenetic placement of †*Hemicalypterus* within †Dapediidae (†Dapediiformes)

The order †Dapediiformes and family †Dapediidae is recovered in this study as a monophyletic group and sister to Ginglymodi (Fig 9) [20, 32, 53]. The †Dapediiformes and Ginglymodi clade is supported by the following four unambiguous synapomorphies in this study: character 17—presence of an independent, ‘splint-like’ quadratojugal; character 63—development of a subrectangular ‘shape of the operculum’, being deeper than long; character 67—suboperculum less than half the depth of the operculum; and character 83—presence of a dorsal ridge of scales. This is consistent with the results of Thies and Waschkewitz [15], which recovered †Dapediiformes as sister to Ginglymodi, however, their phylogenetic analysis included only a single chimaera †*Dapedium* as the sole representative for †Dapediiformes, whereas this study included all putative genera of †Dapediidae [14].

Both methods of phylogenetic reconstruction (parsimony and maximum likelihood) indicate that †*Hemicalypterus weiri* is a member of †Dapediiformes, and that †Dapediiformes forms a monophyletic group with strong bootstrap support in maximum likelihood (BSS = 84) and moderate bootstrap support in parsimony (BSS = 67) (Figs 9 and 10). †*Aetheolepis*, a deep-bodied fish which has previously been hypothesized to belong to either †Semionotidae [38] or †Archaeomaenidae [16], is also inferred in this study to belong to the family †Dapediidae (†Dapediiformes). All other species that have previously been hypothesized to belong to the family †Dapediidae [14] were recovered within †Dapediiformes. Members of †Dapediidae (sensu novo) inferred in this study (Fig 10) include the following eight genera: †*Hemicalypterus*, †*Aetheolepis*, †*Tetragonolepis*, †*Paradapedium*, †*Heterostrophus*, †*Dapedium*, †*Dandya*, and †*Sargodon*. The monophyly of the order †Dapediiformes (Fig 9), including †*Hemicalypterus*, is supported by three unambiguous synapomorphies: character 1—the relative position of the dorsal fin, in †Dapediiformes the dorsal fin originates posterior to the pelvic fins and extends opposite to the anal fin; character 61—exposure of the dorsal limb of the preoperculum, with the dorsal limb being entirely covered or nearly covered by other dermal bones in adults; and character 77—large dorsal fin, with more than 20 rays, which is present in †Dapediiformes.

## Conclusions

This work provides a redescription of the enigmatic taxon †*Hemicalypterus weiri*, with additional morphological information provided based on reexamination of museum specimens as well as examination of newly collected specimens and literature. Using these new morphological insights, †*Hemicalypterus* is placed within a phylogenetic hypothesis of evolutionary relationships, and is recovered as being a member of a monophyletic †Dapediidae and †Dapediiformes. This work will provide a framework for future studies, which will take into account additional lower actinopterygian taxa and novel interpretations of characters as they become available. The phylogenetic relationships of neopterygian ray-finned fishes is still under much consideration and debate, and warrants reexamination of known taxa that have not been considered in an analysis of phylogenetic relationships before.

†*Hemicalypterus weiri* possesses a specialized multicuspid dentition that has not been observed in any other Early Mesozoic ray-finned fishes to date. The presence of isolated, multicuspid teeth belonging to ray-finned fishes of unknown affinity has been recorded in Lower Cretaceous deposits of Xinlong, China ([55]: Fig 4), as well as several locations in Upper Cretaceous deposits in North Africa (e.g., [56, 57]). However, †*Hemicalypterus* is currently the oldest-known representative possessing this type of specialized tooth morphology, and thus possibly represents the oldest example of the evolution of herbivory in the evolutionary history of ray-finned fishes. †*Hemicalypterus* demonstrates that early ray-finned fishes were likely evolving to exploit novel ecological niches at the beginning of the Mesozoic [5]. By reexamining †*Hemicalypterus* and placing it within a hypothesis of evolutionary relationships of early ray-finned fishes, this study provides a framework for understanding the evolution of novel feeding strategies early in the evolutionary history of ray-finned fishes.

## Supporting Information

S1 Table Morphological data matrix of all 54 taxa, 100 characters, used in this study. (PDF)

S1 Text List of phylogenetic characters (Appendix A) used for analysis, and complete list of specimens examined for this study (Appendix B). (DOCX)
