# Supplementary material for: Redescription and Phylogenetic Placement of †Hemicalypterus weiri Schaeffer, 1967 (Actinopterygii, Neopterygii) from the Triassic Chinle Formation, Southwestern United States: New Insights into Morphology, Ecological Niche, and Phylogeny
Source: PLoS One. 2016 Sep 22;11(9):e0163657. doi: 10.1371/journal.pone.0163657 (PMC5033578; doi:10.1371/journal.pone.0163657)
Supplement: S1 Text — (DOCX) [file pone.0163657.s002.docx]

**S1 Text. List of phylogenetic characters (Appendix A) used for analysis, and complete list of specimens examined for this study (Appendix B).**

**Appendix A. List of phylogenetic characters**

1. Relative position of the dorsal fin (López-Arbarello, 2012, modified in Thies and Waschkewitz, 2015): character 1: dorsal fin contained between pelvic and anal fins (0), dorsal fin opposite to anal fin (1), dorsal fin opposite to pelvic fins (2), dorsal fin originates anterior to pelvic fins and extends opposite to anal fin (3), dorsal fin originates posterior to pelvic fins and extends opposite to anal fin (4).
2. Posttemporal fossa (Coates, 1999: character 33): absent (0), present. (1).
3. Forward extension of the exoccipital around the vagus nerve (Olsen and McCune, 1991: character 3): absent (0), present (1).
4. Opistotic (Wiley, 1976: character 6c): present (0), absent (1).
5. Intercalar (Olsen, 1984: character 22): present (0), absent (1).
6. Basisphenoid (Wiley, 1976: character 17b): present (0), absent (1).
7. Sphenotic with small dermal component (Grande, 2010: character 23): absent (0), present (1).
8. Posterior myodome (Wiley, 1976: character 2a): present (0), absent (1).
9. Elongation of the rostral region anterior to the lower jaw symphysis (Grande, 2010: character 4): extends anterior to the dentary symphysis by less than 20% of mandibular length (0), extends well anterior to the dentary symphysis by more than 50% of mandibular length (1).
10. Vomers co-ossified (Olsen, 1984: character 38): absent (0), present (1).
11. Autopalatine missing (Wiley, 1976: character 11b): absent (0), present (1).
12. Ectopterygoid elongate (Wiley, 1976: character 10b): absent (0), present (1).
13. Ectopterygoid participation in palatal surface area (Grande, 2010: character 63): ectopterygoid form half or less of the palatal region (0), ectopterygoid forms the majority of the palatal region (1).
14. Part of dorsal surface of ectopterygoid ornamented and forming part of skull roof (Grande, 2010: character 61): absent (0), present (1).
15. Endopterygoid dentition: present (0), absent (1).
16. Quadrate position in front of the orbit (Wiley, 1976: character 13b): absent (0), present (1).
17. Splint-like quadratojugal (modified from Brito, 1997: character 32): absent (0), present and independent (1), present and partially fused to the quadrate (2), completely fused to the quadrate (3).
18. Symplectic involvement in jaw joint (modified from Grande and Bemis, 1998: character 61): does not articulate with lower jaw (0), distal end articulates with articular bone of lower jaw (1).
19. Ornamentation of the dermal bones of the skull (Grande, 2010: character 2, Grande and Bemis, 1998: character 8): ornamented with tubercles or ridges (0), smooth or very slightly ornamented (1), ornamented with firmly anchored large conical teeth (2).
20. Number of extrascapular bones (modified from Grande and Bemis, 1998: character 49): one pair (0), two pairs (1), three or more pairs (2).
21. Posterior extension of postparietals median to the single pair of laterally placed extrascapular bones: character 21): absent (0), present (1).
22. Relative length of postparietals (parietals) and parietals (frontals) (modified by Thies and Waschkewitz 2015: character 22): length of postparietals less than half but more than one-third the length of parietals (0), length of postparietals about half the length of parietals (1), length of postparietals less than one-third the length of parietals (2), Length of postparietals more than half of the parietals (3).
23. Length of parietals (frontals) (from Jain, 1983; modified from Grande and Bemis, 1998: character 34; character state 2 from Thies and Waschkewitz, 2015): less than 3 times longer than their maximum width (0), 3 or more times longer than their maximum width (1), parietals only slightly longer than their maximum width (2).
24. Parietal (frontal) bones distinctly broader posteriorly, but long and narrow anteriorly (modified from Arratia, 1999: character 188): absent (0), present (1).
25. Antorbital portion of parietal (frontal): broad (0), tapering gradually (1), tubular (2).
26. Parietal (frontal) ethmoidal sagittal lamina: absent (0), present (1).
27. Triangular lateral expansion of antorbital portion of parietal (frontal): absent (0), present (1).
28. Nasals long and narrow: absent (0), present (1).
29. Circumborbital ring (Wiley, 1976: character 9a): supraorbitals do not contact infraorbitals at the anterior rim of the orbit (0), supraorbitals contact infraorbitals, closing the orbit (1).
30. Ventral border of infraorbital series flexes abruptly dorsally at the anterior margin of the orbit: absent (0), present (1).
31. Large supraorbital bones: absent (0), present (1).
32. Most anterior supraorbital bone trapezoidal, longest at ventral margin, and contacting more than one infraorbital bone on ventral margin: absent (0), present (1).
33. A series of toothed infraorbitals bordering the snout (Wiley, 1976: character 3b): absent (0), present (1).
34. Anterior infraorbitals (Olsen and McCune, 1991: character 1): absent (0), present (1).
35. Most anterior infraorbital: lower than or equaling the posterior elements (0), deeper than posterior elements (1).
36. Relative size of the infraorbital bone (or bones) at the posteroventral corner of the orbit: not enlarged (0), enlarged, but do not reach the preoperculum (1), enlarged and reach the preoperculum (2).
37. Shape of the infraorbital bones at the posterior border of the orbit: deeper than long, sometimes almost tubular (0), approximately quadrangular (1), longer than deep, expanded posteriorly (2).
38. Dermosphenotic participation in orbital margin (Grande, 2010: character 16): dermosphenotic reaches orbital margin (0), dermosphenotic does not reach orbital margin (1).
39. Dermosphenotic/sphenotic association (Grande, 2010: character 22): closely associated with each other (i.e. contacting or fused to each other) (0), not in contact with each other (1).
40. Quadrate laterally covered by infraorbital bones: absent (0), present (1).
41. Suborbital bones (Grande and Bemis, 1998: character 7): present (0), absent (1).
42. Number of suborbital bones (modified from Cavin and Suteethorn, 2006: character 4): one (0), two (1), several arranged in one row, which extends anteriad below the orbit (2), mosaic of numerous suborbitals (3), three or four suborbitals arranged in a row, which does not extend anteriad below the orbit (4).
43. Independent of the total number, there is a large suborbital covering almost the whole area between the infraorbital bones and the preoperculum (López-Arbarello, 2012): absent (0), present (1).
44. First and last suborbitals are larger than the other suborbitals: absent (0), present (1).
45. Suborbital series separates preoperculum from dermopterotic: absent (0), present (1).
46. Triangular suborbital lateral to quadrate: absent (0), present (1).
47. Premaxilla with nasal process (modified from Olsen and McCune, 1991: character 4): absent (0), present (1).
48. Premaxillary nasal process forming an external dermal component of the skull roof (Wiley, 1976: character 5b): absent (0), present (1).
49. Supraorbital canal in premaxillary nasal process (Wiley, 1976: character 4b): absent (0), present (1).
50. Length of maxilla: long, extends backwards lateral to the coronoid process of the lower jaw (0), short, does not reach the coronoid process (1), atrophied or absent (2).
51. Depth of maxilla: shallow (0), deep (1).
52. Supramaxilla (Wiley, 1976: character 3a): absent (0), present, single bone (1), present, two bones (2).
53. Maxillary teeth (Cavin, 2010: character 30): present (0), absent (1).
54. Plicidentine (Wiley, 1976: character 27b): absent (0), present (1).
55. Tritoral dentition (from Jain, 1983): absent (0), moderately tritoral (1), extremely tritoral (2).
56. Well-developed posteroventral process of the dentary (from Thies, 1989): absent (0), present (1).
57. Double row of teeth in dentary (modified from Grande, 2010: character 39): absent (0), present (1).
58. Mandibular symphysis very deep (from Jain, 1983): absent (0), present (1).
59. Extent of teeth on denture (excluding coronoid tooth plates) (Grande, 2010: character 56): tooth row extends over a third the length of dentary (0), tooth row is present on only the anterior one third or less of dentary (1).
60. Shape of preoperculum: dorsoventrally elongated without anteroventral arm (0), crescent-shaped (1), L-shaped (2).
61. Exposure of dorsal limb of preoperculum (Grande, 2010: character 73): mostly exposed forming a significant part of the ornamented lateral surface of the skull anterior to the operculum (0), entirely covered or nearly entirely covered by other dermal bones in adults (1).
62. Posterior border of preoperculum notched ventrally: absent (0), present (1).
63. Shape of the operculum: subrectangular, deeper than long (0), rounded to quadrate, approximately as deep as long (1), tapering anteroventrally (2).
64. Suboperculum with well-developed ascending process: absent (0), present (1).
65. Shape of ascending process of the suboperculum: robust, with broad base and rounded distal end (0), slender, tapering dorsad (1).
66. High ascending process of the suboperculum: less than or equal to half of the length of the dorsal border of the bone (0), more than half of the length of the dorsal border of the bone (1).
67. Suboperculum less than half the depth of the operculum: absent (0), present (1).
68. Interoperculum (modified from Wiley, 1976: character 10a): absent (0), present (1).
69. Size of interoperculum: large, approximately as long as the ventral arm of the preoperculum (0), small, remote from mandible (1).
70. Gular plate (modified from Olsen and McCune, 1991: character 8): double (0), single (1), absent (2).
71. Opistocoelous vertebrae (Wiley, 1976: character 26b): absent (0), present (1).
72. Knob-like anteroventral process of posttemporal: absent (0), present (1).
73. Supracleithrum with a concave articular facet for articulation with the posttemporal (Grande, 2010: character 93): absent (0), present (1).
74. Series of denticles along the ridge between the branchial and lateral surfaces of the cleithrum (from Bartram, 1977): absent (0), one or two rows (1), several rows (2).
75. Fringing fulcra on pectoral fin: present (0), absent (1).
76. Fringing fulcra on pelvic fin: present (0), absent (1).
77. Large dorsal fin, with more than 20 rays: absent (0), present (1).
78. Large basal fulcra in the dorsal and anal fins: absent (0), present (1).
79. Scale-like ray at the dorsal margin of the caudal fin (from Bartram, 1977): absent (0), present (1).
80. A constant number of exactly eight lepidotrichia in the lower, non-axial lobe of the tail (from Bartram, 1977): absent (0), present (1).
81. A constant number of exactly six lepidotrichia in the lower, non-axial lobe of the tail: absent (0), present (1).
82. Body lobe scale row (modified from Lombardo and Tintori, 2008): absent (0), present, with additional incomplete row (1), present, without additional incomplete row (2).
83. Dorsal ridge of scales (modified from Olsen and McCune, 1991: character 17): inconspicuous (0), conspicuous, with a low spine (1), conspicuous, with a deep spine (2), toothed or spinose (3).
84. Scales of the body with a strong posteriorly directed spine: absent (0), present (1).
85. Vertical peg-and-socket articulation: present (0), reduced or absent (1).
86. Longitudinal articulation of the scales of the body: absent (0), single (1), double (2).
87. Posttemporal penetration by lateral line canal (Grande, 2010: character 91): present (0), absent (1).
88. Supraorbital sensory canal in postparietal (parietal) (modified from Wiley, 1976): supraorbital canal penetrates postparietals at the central portion of these bones (0), supraorbital canal running almost on the lateral rim of the postparietals (1), supraorbital canal does not penetrate the postparietals (2).
89. Orbital canal (sensory canal present in supraorbital bones): absent (0), present (1).
90. Deep groove housing the middle pit line in dermopterotic and postparietal: absent (0), present (1).
91. Premaxillary teeth: unicuspid (0), bicuspid (1), multicuspid (2).
92. Ventral Ridge Scales: absent (0), present (1).
93. Scale thickness posterior half of body (striate squamation): no reduction in thickness (0), reduced in thickness or lost (1).
94. Body deeply fusiform to nearly circular: absent (0), present (1).
95. Dorsal and anal fins hem-like: absent (0), present (1).

96. Suprapreopercular bone (from Bermudez-Rochas and Poyato-Ariza 2014): 0. Absent (0), present (1).

97. Number of infraorbitals anterior to the orbit (from Bermudez-Rochas and Poyato-Ariza 2014): none (0), one (1), two (2), three (3), four (4), five or more (5).

1. Tube-like, canal bearing, anterior arm of the antorbital (from Arratia 2013): absent (0), present (1).
2. Two vertebral centra fused into occipital condyle in adults (from Arratia 2013): absent (0), present (1).
3. Arrangement of hypurals and caudal fin rays (from Arratia 2013, modified from Grande and Bemis 1998 character 46): each hypural normally articulated with one caudal ray (0), each hypural normally articulated with a few caudal rays (1), hypural plate articulated with many rays (2).

**References Cited in Appendix A**

Arratia G (1999) The monophyly of Teleostei and stem-group teleosts Consensus and disagreements. In Arratia G, Schultze H-P editors. Mesozoic Fishes 2—Systematics and the Fossil Record. München, Verlag Dr. F. Pfeil. 265–334.

Arratia G (2013) Morphology, taxonomy, and physiology of Triassic pholidophorid fishes (Actinopterygii, Teleostei). Soc Vertebr Paleontol Mem 13: 1–138.

Bartram AWH (1977) The Macrosemiidae, a Mesozoic family of holostean fishes. Bull Brit Mus (Nat Hist) Geo 29: 137–234.

Bermúdez-Rochas D, Poyato-Ariza FJ (2014) A new semionotiform actinopterygian fish from the Mesozoic of Spain and its phylogenetic implications. J Sys Palaeontol 13: 265–285.

Brito PM (1997) Révision des Aspidorhynchidae (Pisces, Actinopterygii) du Mésozoique: ostéologie, relations phylogéné tiques, donné es environmentales et biogéographiques. Geodiversitas 19: 681–772.

Cavin L, Suteethorn V (2006) A new semionotiform (Actinopterygii, Neopterygii) from Upper Jurassic-Lower Cretaceous deposits of northeast Thailand, with comments on the relationships of semionotiforms. Palaeontology 49: 339–353.

Coates MI (1999) Endocranial preservation of a Carboniferous actinopterygian from Lancashire, UK, and the interrelationships of primitive actinopterygians. Phil Trans R Philos Soc London B 345: 435–462.

Grande L (2010) An empirical synthetic pattern study of gars (Lepisosteiformes) and closely related species, based mostly on skeletal anatomy. The resurrection of Holostei. Am Soc Ichthyols Herpetol Spec Publ 6: 1–871.

Grande L, Bemis WE (1998) A comprehensive phylogenetic study of amiid fishes (Amiidae) based on comparative skeletal anatomy. An empirical search for interconnected patterns of natural history. Soc Vert Paleontol Mem 4: 1–690.

Jain SL (1983) A review of the genus Lepidotes (Actinopterygii: Semionotiformes) with special reference to the species from the Kota Formation (Lower Jurassic), India. J Palaeontol Soc India 28: 7–42.

Lombardo C, Tintori A (2008) A new semionotid fish (Actinopterygii: Osteichthyes from the Late Triassic of northern Italy. In Arratia G, Schultze H-P, Wilson MVH editors. Mesozoic Fishes 4 — Homology and Phylogeny. Verlag Dr. Friedrich Pfeil, München, Germany. 129–142.

López-Arbarello A (2012) Phylogenetic Interrelationships of Ginglymodian Fishes (Actinopterygii: Neopterygii). PLOS ONE 7(7): e39370. doi: 10.1371/journal.pone.0039370.

Olsen PE (1984) The skull and pectoral girdle of the parasemionotid fish *Watsonulus eugnathoides* from the Early Triassic Sakamena Group of Madagascar, with comments on the relationships of holostean fishes. J Vertebr Paleontol 4(3): 481–499.

Olsen PE, McCune AR (1991) Morphology of the †*Semionotus elegans* species group from the Early Jurassic part of the Newark Supergroup of eastern North America with comments on the family Semionotidae (Neopterygii). J Vertebr Paleontol 11(3): 269–292.

Thies D (1989) The braincase and the brain of *Tetragonolepis semicincta* Bronn 1830 (Actinopterygii, †Semionotiformes). Palaeontogr Abt A 209: 1–32.

Thies D, Waschkewitz J (2015) Redescription of *Dapedium pholidotum* (Agassiz, 1832) (Actinopterygii, Neopterygii) from the Lower Jurassic Posidonia Shale, with comments on the phylogenetic position of *Dapedium* Leach, 1822. J Sys Palaeontol. doi 10.1080/14772019.2015.1043361.

Wiley EO (1976) The phylogeny and biogeography of fossil and recent gars (Actinopterygii: Lepisosteidae). Univ Kansas Misc Pub 64: 1–111.

**Appendix B. Specimen Material Examined**

***Institutional abbreviations.* AMNH**, American Museum of Natural History, New York; **AMS**, Australian Museum, Sydney; **FMNH**, Field Museum of Natural History, Chicago; **SGDS**, St. George Dinosaur Discovery Site at Johnson Farm, St. George, Utah; **UMNH**, Natural History Museum of Utah, Salt Lake City, Utah.

*Amia fragosa*: FMNH PF 10299

*Amia pattersoni*: FMNH PF 14343

*Amia scutata*: FMNH PF 13108, PF 14313, PF 15345, PF 15347

*Araripelepidotes temnurus*: AMNH 11813, 19067; FMNH PF 11835, PF 11849, PF 11852–11853, PF 14043, PF 14349

*Aetheolepis mirabilis*: AMS MF260, MF262 (photographs only)
*Dapedium leachii*: USNM V 16494

*Dapedium pholidotum*: FMNH P 25056, UC 2056; AMNH 7538, USNM V 4922

*Dapedium politum*: USNM V 1922–1923
*Dapedium punctatum*: FMNH PF 25433; USNM V 16108
*Hemicalypterus weiri*: AMNH 5709–5718, UMNH VP 19419, VP 22904; USNM V 23422, V 23424, V 23425 (holotype), V23427–23429

*Heterostrophus phillipsi*: BGS GSM113113a–c (Holotype; photographs only)

*Lepidotes elvensis*: FMNH P 25095, PF 5367

*Lepidotes notopterus*: FMNH UF 539

*Lepidotes oblongus*: FMNH UC 2138

*Lepidotes* sp.: FMNH P 25351, P 25588, PF 12546, PF 15470

*Leptolepides sprattiformis*: FMNH P 25307, UC 2118

*Cavenderichthys talbragarensis*: FMNH PF 5998

*Lophionotus chinleana*: AMNH 5682-5683; UMNH VP 19415, VP 19417–19418

*Lophionotus kanabensis*: AMNH 8870 (holotype), 8871; USNM V 18399

*Lophionotus sanjuanensis*: AMNH 5679, 5680, 5684, 5685, 5690; USNM V 23417, V 23420; UMNH VP 19419–19421

*Lophionotus* sp. UMNH VP 19413–19419, VP 29416

*Dorsetichthys bechei*: FMNH UC 2137

*Pholidophoroides limbatus*: FMNH UC 2119

*Platysomus lacovianus*: AMNH 7400 (type)

*Platysomus gibbosus*: USNM V 21805

*Sargodon tomicus*: FMNH UC 952, UF 953;

*Semionotus agassizii*: AMNH 8459

*Semionotus capensis*: AMNH 8828, 8829, 19702; FMNH P 25053–25056

*Semionotus elegans*: FMNH P 12751, UC 2060, UF 551

*Semionotus fultus*: FMNH UF 958; MCZ 6152

*Semionotus micropterus*: FMNH PF 13104, UC 2059, UF 37

*Semionotus tenuiceps*: FMNH P 12548, P 25049, PF 13105, PF 25050–25052, UF 431

*Semionotus* sp.: AMNH 5681, 5686–5689, 5691–5696, 5698–5699, 5702–5703, 5705–5707, 18970–18972; FMNH PF 5732, PF 13106, PF 15156, UC 2006, UF 452–458, UF 957; SGDS 886, 894, 1059, 1237, 1241, 1314

*Scheenstia ‘Lepidotes’ maximus*: AMNH 13097; FMNH P 25587, UF 950

*Teoichthys kallistos*: USNM 460260

*Tetragonolepis semicincta*: AMNH 7541; FMNH UF 36; USNM V 279855

*Watsonulus eugnathoides*: FMNH PF 4256
