# Supplementary material for: Redescription and Phylogenetic Placement of †Hemicalypterus weiri Schaeffer, 1967 (Actinopterygii, Neopterygii) from the Triassic Chinle Formation, Southwestern United States: New Insights into Morphology, Ecological Niche, and Phylogeny
Source: PLoS One. 2016 Sep 22;11(9):e0163657. doi: 10.1371/journal.pone.0163657 (PMC5033578; doi:10.1371/journal.pone.0163657)
Supplement: S1 Table — (PDF) [file pone.0163657.s001.pdf]

S1 Table. Matrix of Morphological Characters

	1	2	3	4	5	6	7	8	9	10	11	12	13	14	15	16	17	18	19	20	21	22	23	24	25	26	27	28	29	30	31	32	33	34	35	36	37	38	39	40	41	42	43	44	45	46	47	48	49	50
Perleidus	0	1	0	0	0	0	?	0	0	?	?	0	?	?	?	0	0	?	0	0	0	0	0	0	0	0	0	0	0	0	0	0	0	0	?	0	0	0	0	0	0	0	0	0	0	0	0	?	?	0
Watsonulus	0	1	0	?	0	?	0	0	0	0	0	0	0	0	0	0	0	1	0	0	0	2	0	0	0	0	0	0	1	0	0	0	0	0	?	1	0	0	0	0	1	?	?	?	?	?	1	0	0	0
Amia	3	1	0	1	0	0	1	0	0	0	0	0	0	0	0	0	0	1	0	0	0	1	0	0	0	0	0	0	?	0	?	?	0	0	?	1	2	0	0	0	1	?	?	?	?	?	1	0	0	0
Leptolepis	?	?	0	0	0	0	0	0	0	1	?	0	0	0	?	0	3	0	0	0	0	2	1	1	0	0	0	?	0	0	0	0	0	0	?	2	2	0	0	0	0	0	1	0	0	0	0	?	?	0
Pholidophorus	2	1	0	0	0	0	0	0	0	1	?	0	0	0	?	0	3	0	0	0	0	2	0	1	0	0	0	0	0	0	0	0	0	0	?	2	2	0	0	0	0	0	1	0	0	0	0	?	?	0
Siemensichthys	2	?	?	?	?	?	0	0	0	1	0	0	?	?	?	0	3	0	0	0	0	2	0	0	0	0	0	?	?	0	?	?	0	0	?	2	2	?	0	0	1	?	?	?	?	?	0	?	?	0
Lepisosteus	1	0	1	1	1	1	1	1	0	0	1	1	1	1	0	1	1	0	0	1	0	0	1	0	0	0	0	0	1	1	1	1	1	1	0	2	1	0	1	1	0	3	0	0	0	0	1	1	1	2
Atractosteus	1	0	0&1	1	1	1	1	1	0	0	1	1	1	1	0	1	1	0	0	2	0	0	1	0	0	0	0	0	1	1	1	1	1	1	0	2	1	0&1	1	1	0	3	0	0	0	0	1	1	1	2
Masillosteus	1	?	?	?	?	?	1	?	0	?	?	0	1	0	?	1	?	0	0	1	0	0	0	0	0	0	0	0	1	1	1	1	0	1	0	2	2	0&1	1	1	0	3	0	0	0	0	1	1	?	2
Obaichthys	1	0	1	1	1	1	1	1	1	0	?	1	1	0	?	1	0	0	2	1	0	0	0	0	0	0	0	0	1	1	0	?	0	1	0	2	1	1	0	1	0	3	0	0	0	0	1	1	1	1
Dentilepisosteus	1	0	1	1	1	1	1	1	1	0	1	1	1	0	?	1	1	0	2	1	0	0	1	0	0	0	0	0	1	1	0	1	0	1	?	2	2	1	0	1	0	3	0	0	0	0	1	1	1	1
Pliodetes	2	?	?	?	?	?	0	?	1	0	?	?	?	?	?	0	1	?	2	1	0	1	0	0	0	0	0	0	1	1	1	1	0	1	0	1	2	0	0	1	0	3	0	0	0	0	1	1	1	1
Araripelepidotes	2	1	1	1	1	0	0	0	?	?	?	?	?	?	?	0	?	?	1	1	0	1	0	0	0	0	0	?	?	?	1	?	0	1	0	2	2	0	0	1	0	4	0	0	0	0	?	0	?	1
Isanichthys	1	?	?	?	?	?	0	?	0	?	?	?	?	?	?	0	?	?	0	1	0	0	0	0	0	0	0	?	1	1	1	1	0	1	1	0	2	1	?	0	0	2	0	0	0	0	?	?	?	0
Scheenstia maximus	0	?	?	?	?	?	0	?	0	1	0	0	?	?	?	0	?	?	0	2	0	0	0	0	0	0	0	?	1	1	1	1	0	1	0	0	2	0	0	0	0	2	0	1	0	0	1	0	0	?
Scheenstia laevis	0	?	?	?	?	?	0	?	0	?	?	?	?	?	?	0	?	?	0	2	0	0	1	0	0	0	0	?	1	1	1	1	0	1	0	0	2	0	0	0	0	2	0	1	0	0	?	0	?	1
Scheenstia mantelli	0	1	1	1	1	0	0	?	0	1	?	0	?	?	?	0	?	?	0	2	0	0	1	0	0	0	0	?	1	1	1	1	0	1	0	0	2	0	0	0	0	2	0	1	0	0	1	0	?	1
Scheenstia zappi	0	?	?	?	?	?	0	?	0	?	0	0	?	?	?	0	1	0	0	2	0	0	0	0	0	0	0	0	1	1	1	0	0	1	0	0	1	0	0	0	0	2	0	1	0	0	1	0	0	?
Lepidotes semiserratus	0	?	1	1	?	0	0	0	0	1	?	0	0	0	?	0	1	0	0	0	0	0	0	0	0	0	0	0	1	?	1	1	0	1	1	0	1	0	0	0	0	2	0	0	0	0	1	0	?	0
Lepidotes gigas	0	?	?	?	1	?	0	?	0	?	0	0	0	0	?	0	1	0	1	0	0	0	0	0	0	0	0	0	1	0	1	1	0	1	1	0	1	0	0	0	0	2	0	0	0	0	1	0	0	0
Lepidotes elvensis	?	?	?	?	?	?	0	?	0	?	?	0	0	0	?	0	?	?	1	0	?	0	0	0	0	?	0	0	1	1	1	1	0	1	1	0	1	0	?	0	0	2	0	0	0	0	1	0	?	0
Neosemionotus	0	?	?	?	?	?	0	?	0	0	?	?	?	?	?	0	?	0	1	2	0	0	0	0	0	0	0	1	1	0	1	0	0	1	0	0	1	0	0	0	0	4	0	0	0	0	1	0	0	0
Callipurbeckia minor	0	?	1	1	1	0	0	?	0	?	?	0	0	0	?	0	?	0	0	0	0	2	1	0	1	0	0	1	1	0	0	0	0	1	0	0	0	0	0	0	0	1	1	0	0	0	1	0	0	0
Tlayuamichin	0	?	?	?	?	?	1	?	0	0	?	0	?	?	1	0	?	0	0	1	0	2	1	0	1	0	0	1	0	0	0	0	0	1	0	0	0	0	0	0	0	4	0	0	1	0	1	0	0	0
Macrosemimimus fegerti	0	?	?	?	?	0	0	?	0	1	?	0	0	0	?	0	1	0	1	?	1	0	1	0	2	1	0	1	0	0	0	0	0	1	0	0	0	0	0	0	0	1	1	0	0	0	1	0	0	0
Macrosemimimus lennieri	0	1	1	1	1	0	0	0	0	1	0	0	0	0	1	0	1	0	0	0	0	0	1	0	2	1	0	?	0	0	1	0	0	1	0	0	0	0	0	0	0	1	1	0	0	0	1	0	0	0
Semionotus bergeri	0	?	?	?	?	?	1	?	0	?	?	0	?	?	?	0	?	0	0	0	0	0	1	0	1	0	1	?	1	0	0	0	0	1	0	1	0	0	0	0	0	0	1	0	0	0	1	0	0	?
Semionotus elegans	0	?	1	1	1	?	1	0	0	0	0	0	0	0	?	0	1	0	1	0	0	0	1	0	1	0	1	1	1	0	0	0	0	1	0	0	0	0	0	0	0	0	1	0	0	0	1	0	0	0
Semionotus capensis	0	?	?	?	?	?	1	?	0	?	0	0	0	0	?	0	1	0	1	1	0	0	1	0	1	0	1	1	0	0	0	0	0	1	0	1	0	0	0	0	0	0	1	0	0	0	1	0	0	0
Paralepidotus	3	?	?	?	?	?	0	?	0	0	?	0	?	?	?	0	1	?	0	0	0	2	1	0	1	0	0	1	0	0	0	0	0	1	0	1	0	0	0	0	0	0	1	0	0	0	?	0	?	?
Semiolepis	0	?	?	?	?	?	?	?	0	?	?	0	0	0	?	0	1	?	0	2	0	2	1	0	1	0	0	?	0	0	0	0	0	1	0	2	0	0	0	0	0	0	1	0	0	0	1	0	0	0
***Macrosemius* spp**	3	?	1	1	1	1	0	0	0	0	?	0	0	0	1	0	2	0	1	?	1	2	1	0	2	0	0	1	0	0	?	0	0	1	0	0	0	0	0	0	1	?	?	?	?	?	1	0	0	0
***Notagogus* spp**	3	?	?	?	?	?	?	?	0	?	?	?	?	?	?	0	2	0	1	?	1	2	1	0	2	0	0	?	0	0	0	0	0	1	0	0	0	?	0	0	1	?	?	?	?	?	1	0	0	0
***Propterus* spp**	3	?	?	?	?	1	0	?	0	0	?	?	?	?	1	0	1	0	1	?	1	2	1	0	2	0	0	1	0	0	0	0	0	1	0	0	0	0	0	0	1	?	?	?	?	?	1	0	0	0
Sangiorgioichthys sui	0	?	?	?	?	?	0	?	0	?	0	0	0	0	?	0	1	0	0	1	0&1	0	1	0	0	0	0	1	0	0	0	0	0	1	0	1&2	0	0	0	0	0	2	1	0	0	1	1	0	0	0
Sangiorgioichthys aldae	0	?	?	?	?	?	0	?	0	?	?	?	0	0	?	?	?	?	0	0	0	0	1	0	0	0	0	1	0	0	0	0	0	1	0	2	0	0	0	0	0	2	0	0	1	1	1	0	0	0
Luoxiongichthys	3	?	?	?	?	?	?	?	0	?	?	0	0	0	?	0	1	?	0	?	?	0	1	0	0	0	1	1	1	0	0	0	0	1	0	1	0	0	?	1	0	2	0	0	0	0	?	0	?	0
Lophionotus sanjuanensis	0	?	?	?	?	?	0	?	0	?	?	0	0	0	?	0	1	?	0	0	0	0	0	0	0	0	1	?	1	1	0	0	0	1	0	2	0	0	?	1	0	0	1	0	0	0	1	0	0	0
Lophionotus chinleana	0	?	?	?	?	?	0	?	0	?	?	?	?	0	?	0	1	0	1	0	0	0	0	0	0	0	1	?	1	1	0	0	0	1	0	2	0	0	?	1	0	0	1	0	0	0	1	0	0	?
Lophionotus kanabensis	0	?	?	?	?	?	0	?	0	?	0	0	0	0	?	0	1	?	1	0	0	0	0	0	1	0	1	?	?	?	?	?	0	1	?	2	?	0	?	0	0	0	1	0	0	0	1	0	0	?
Camerichthys	?	?	?	?	?	?	1	?	0	?	?	?	?	?	?	0	?	0	0	?	?	?	1	0	1	0	0	1	1	0	0	1	0	1	2	0	1	0	0	0	0	2	1	1	0	0	1	0	0	0
Archaeosemionotus connectens	1	?	?	?	?	?	0	?	0	?	?	?	?	0	?	0	?	?	0	?	?	?	0	0	0	?	0	?	1	0	0	0	0	?	?	1	1	?	?	?	1	4	1	1	0	?	1	?	?	1
Hemicalypterus weiri	4	?	?	?	?	?	0	?	0	?	?	0	0	0	?	0	1	?	0	1	0	3	0	1	1	?	0	?	1	?	0	0	0	0	?	1	1	0	0	?	0	2	1	1	0	?	1	?	?	0
Sargodon tomicus	4	?	?	?	?	?	?	?	0	1	?	0	?	0	?	0	?	?	0	?	0	3	0	0	0	?	0	0	1	0	0	0	0	0	?	0	1	0	?	0	0	2	0	0	0	1	1	?	?	0
Dandya ovalis	4	?	?	?	?	?	?	?	0	?	?	?	?	0	?	0	?	?	0	2	0	1	0	0	0	?	0	0	?	0	?	?	0	?	?	0	?	?	?	0	0	2	0	0	1	1	0	?	?	0
Dapedium caelatum	4	?	?	?	0	?	?	0	0	0	1	?	?	?	?	0	?	0	0	2	0	3	2	0	0	?	0	0	0	0	0	0	0	0	?	?	2	0	0	0	0	2	0	0	0	0	0	?	?	0
Dapedium stollorum	4	?	?	?	?	?	?	?	0	?	1	?	?	0	?	0	?	?	0	2	0	3	2	0	0	?	0	0	0	0	0	0	0	0	?	1	2	0	?	0	0	2	0	1	1	0	0	?	?	0
Dapedium punctatum	4	?	?	?	0	?	?	?	0	0	1	?	?	0	?	0	?	0	0	2	0	3	2	0	0	?	0	0	1	0	0	0	0	0	?	1	0	0	?	0	0	2	0	0	1	0	0	?	0	0
Dapedium pholidotum	4	?	?	?	?	?	?	?	0	?	1	?	?	0	?	?	?	0	0	2	0	3	2	0	?	?	0	0	1	0	0	0	0	0	?	1	2	0	0	0	0	2	0	0	1	0	?	?	?	0
Paradapedium egertoni	4	?	?	?	?	?	?	?	0	?	?	0	?	0	?	0	?	?	0	0	0	3	0	0	0	?	0	0	?	0	?	?	0	?	?	0	0	?	?	?	0	2	0	0	1	0	?	?	?	?
Tetragonolepis oldhami	4	?	?	?	?	?	0	?	0	?	?	?	?	0	?	0	?	?	0	0	0	3	0	0	0	?	0	0	1	0	0	?	0	0	?	?	?	?	?	0	0	2	0	1	0	?	?	?	?	?
Tetragonolepis semicincta	4	?	?	?	?	0	0	?	0	0	0	?	?	0	?	0	1	?	0	0	0	3	0	0	0	?	0	0	1	0	0	0	0	0	?	0	1	0	?	0	0	2	0	1	0	0	1	1	0	0
Aetheolepis	4	?	?	?	?	?	?	?	?	?	?	?	?	?	?	0	?	?	0	?	?	?	?	?	?	?	?	?	?	?	?	?	0	?	?	?	?	?	?	?	?	?	?	?	?	?	?	?	?	?
Kyphosichthys grandei	0	?	?	?	?	?	?	?	0	?	?	?	?	0	?	0	1	?	0	1	0	2	1	0	1	?	?	0	?	0	0	?	0	1	1	2	1	0	?	1	0	1	0	0	1	0	1	?	?	0
Heterostrophus phillipsi	4	?	?	?	?	?	?	?	0	?	?	0	?	0	?	0	?	?	0	1	?	2	0	0	0	?	0	0	0	1	0	?	0	1	0	1	?	0	?	?	0	2	0	0	1	0	0	?	?	0

	51	52	53	54	55	56	57	58	59	60	61	62	63	64	65	66	67	68	69	70	71	72	73	74	75	76	77	78	79	80	81	82	83	84	85	86	87	88	89	90	91	92	93	94	95	96	97	98	99	100
Perleidus	0	0	0	0	1	0	0	0	0	0	0	0	1	0	?	?	0	0	?	0	0	0	0	0	0	0	0	0	?	0	0	0	0	0	0	0	0	0	0	0	0	0	?	0	0	0	0	0	?	?
Watsonulus	0	1	0	0	0	1	0	0	0	0	0	0	1	0	?	?	0	1	1	1	0	0	0	0	0	0	0	0	?	0	0	0	0	0	0	0	0	0	0	0	0	0	?	0	0	0	0	1	?	?
Amia	0	1	0	0	0	0	0	0	0	1	0	0	1	1	0	0	1	1	1	1	0	0	0	0	1	1	1	?	0	?	?	?	?	0	?	?	0	2	0	0	0	0	?	0	0	0	0	1	1	0
Leptolepis	0	2	0	0	0	1	0	0	?	1	0	0	2	0	?	?	0	1	0	0	0	0	0	0	1	1	0	0	0	?	?	?	0	0	?	?	0	0	0	0	0	?	?	0	0	0	0	0	0	1
Pholidophorus	0	2	0	0	0	1	0	0	0	1	0	0	2	0	?	?	0	1	?	0	0	0	0	0	1	1	0	0	0	?	?	?	0	0	0	0	0	0	0	0	0	?	?	0	0	0	0	0	?	1
Siemensichthys	0	1	0	0	0	1	0	0	?	1	0	0	2	0	?	?	0	1	?	0	0	0	0	0	0	0	0	0	0	?	?	0	0	0	0	0	0	0	0	0	0	?	?	0	0	0	?	0	0	?
Lepisosteus	?	0	?	1	0	0	1	0	0	2	1	0	1	1	0	0	0	0	?	2	1	0	1	0	0	0	0	0	1	0	1	0	0	0	1	1	1	2	1	1	0	0	0	0	0	0	4	1	1	0
Atractosteus	?	0	?	1	0	0	1	0	0	2	1	0	1	1	0	0	0	0	?	2	1	0	1	0	0	0	0	0	0	0	1	0	0	0	1	1	1	2	1	1	0	0	0	0	0	0	4	1	?	?
Masillosteus	?	0	?	1	1	0	0	0	1	2	1	0	1	1	0	0	0	0	?	2	1	0	1	0	0	0	0	0	0	0	1	0	0	0	1	2	1	2	0	1	0	0	0	0	0	0	2	1	?	?
Obaichthys	0	?	0	0	0	0	0	0	1	2	0	0	1	1	1	0	1	1	1	2	1	0	0	0	0	0	0	0	?	?	?	?	0	1	0	1	?	2	0	0	0	0	0	0	0	0	5	1	1	0
Dentilepisosteus	0	1	0	0	0	1	0	0	1	2	0	0	1	1	0	0	0	1	1	2	1	0	0	?	0	0	0	0	0	0	1	0	0	1	1	1	0	2	0	1	0	0	0	0	0	0	5	1	?	?
Pliodetes	0	0	1	0	0	0	0	0	?	2	0	0	1	1	1	0	0	0	?	2	0	0	1	?	0	0	0	0	?	?	?	?	0	1	0	1	0	2	0	0	0	0	0	0	0	0	?	1	?	?
Araripelepidotes	0	0	1	?	0	0	0	0	?	2	0	0	1	1	1	0	1	1	1	2	0	0	1	0	0	0	0	0	0	0	0	0	0	1	0	2	0	2	0	0	0	0	0	0	0	0	1	1	?	?
Isanichthys	0	1	0	0	?	1	0	0	0	1	0	0	0	1	1	0	1	1	0	2	0	0	?	?	?	?	?	0	?	?	?	?	0	0	?	?	0	1	0	0	0	0	0	0	0	0	2	0	?	?
Scheenstia maximus	?	?	?	0	2	1	0	1	0	1	0	0	0	1	0	0	1	1	0	2	?	1	1	?	0	0	0	0	0	0	0	0	0	0	?	2	0	?	1	?	0	0	0	0	0	0	2	?	?	?
Scheenstia laevis	1	?	1	0	2	1	0	1	?	1	0	0	0	1	0	0	1	1	0	?	?	1	1	?	0	0	?	?	?	?	?	?	0	0	?	?	0	1	1	1	0	0	0	0	0	0	3	?	?	?
Scheenstia mantelli	1	1	1	0	2	1	0	1	?	1	0	0	0	1	1	0	1	1	0	2	0	1	1	1	0	0	0	0	0	0	0	0	0	0	0	2	0	1	?	1	0	0	0	0	0	0	3	?	?	?
Scheenstia zappi	?	?	?	0	2	1	0	0	0	1	0	0	0	1	1	0	1	1	0	2	?	1	1	2	?	0	0	0	0	0	0	0	0	0	?	?	0	1	1	1	0	0	0	0	0	0	3	1	?	?
Lepidotes semiserratus	0	1	0	0	1	1	0	0	?	1	0	0	0	1	1	0	1	1	0	2	0	?	?	2	0	0	?	?	?	?	?	?	0	0	1	2	0	1	?	1	0	0	0	0	0	0	3	?	?	?
Lepidotes gigas	0	1	0	0	0	1	0	0	0	1	0	0	0	1	1	0	1	1	0	2	0	0	1	2	0	?	0	0	0	0	0	0	0	0	1	2	0	1	?	1	0	0	0	0	0	0	2	?	?	?
Lepidotes elvensis	0	1	0	?	0	1	0	0	0	1	0	0	0	0	?	?	1	1	0	?	?	0	1	?	0	?	?	?	?	?	?	?	0	0	?	?	0	?	0	1	0	0	?	0	0	0	2	?	?	?
Neosemionotus	0	1	0	0	0	1	0	0	0	1	0	0	0	1	1	0	1	1	1	2	?	0	1	?	?	0	0	1	1	0	0	0	1	0	?	?	0	0	?	0	0	0	0	0	0	0	2	1	?	?
Callipurbeckia minor	1	1	1	0	1	1	0	0	0	1	0	0	0	1	1	1	1	1	0	2	0	0	1	2	0	0	0	1	0	1	0	1	1	0	0	2	0	1	?	0	0	0	0	0	0	0	2	?	?	?
Tlayuamichin	1	1	1	0	1	1	0	0	0	1	0	0	0	1	1	1	1	1	0	2	0	0	1	2	0	0	0	1	0	1	0	1	1	0	?	2	0	0	1	0	0	0	0	0	0	0	5	1	?	?
Macrosemimimus fegerti	1	1	1	0	1	1	0	0	0	1	0	0	0	1	1	1	1	1	0	2	?	0	1	2	0	0	0	0	0	1	0	1	0	0	0	?	0	?	1	0	0	0	0	0	0	0	4	0	?	?
Macrosemimimus lennieri	0	1	1	0	2	1	0	1	0	1	0	0	0	1	1	1	1	1	0	2	?	0	1	2	0	0	0	0	?	?	?	?	0	0	?	2	0	0	?	0	0	0	0	0	0	0	3	?	?	?
Semionotus bergeri	?	?	0	0	0	?	0	0	0	1	0	0	0	1	1	0	1	1	0	2	?	0	?	1	0	0	0	1	?	1	0	?	2	0	0	1	0	0	0	0	0	0	0	0	0	0	?	1	?	?
Semionotus elegans	0	1	0	0	0	1	0	0	0	1	0	0	0	1	1	0	1	1	0	2	0	0	0	1	0	0	0	1	?	1	0	1	2	0	?	?	0	0	0	0	0	0	0	0	0	0	1	1	?	0
Semionotus capensis	0	1	?	?	0	1	0	0	0	1	0	0	0	1	1	0	1	1	0	2	?	0	1	?	0	0	0	1	1	1	0	1	2	0	0	1	0	0	0	0	0	0	0	0	0	0	?	1	?	?
Paralepidotus	?	?	1	?	1	?	0	0	0	1	0	0	0	1	1	1	1	1	0	2	0	?	?	1	0	0	1	0	1	0	0	1	0	0	0	1	0	?	?	0	0	0	?	0	0	0	3	1	?	?
Semiolepis	0	1	0	0	1	1	0	0	0	1	0	0	0	1	1	1	1	1	1	2	0	0	1	1	0	0	0	0	1	0	0	1	1	0	0	1	0	?	?	?	0	0	?	0	0	0	3	1	?	?
***Macrosemius* spp**	0	0	0	0	1	0	0	0	0	1	0	1	0	1	1	0	1	1	1	2	0	0	0	1	1	1	1	?	1	1	0	?	?	0	0	0	0	2	?	0	0	0	?	0	0	0	3	1	?	?
***Notagogus* spp**	0	0	0	0	0	0	0	0	0	1	0	1	0	1	1	0	1	1	1	2	0	0	0	1	0&1	1	1	0	0	1	0	?	0	0	0	0	0	2	0	0	0	0	?	0	0	0	3	1	?	?
***Propterus* spp**	0	0	0	0	0	0	0	0	0	1	0	1	0	1	1	1	1	1	1	2	0	0	0	1	1	0	1	0	1	1	0	1	0	0	0	0	0	2	0	0	0	0	?	0	0	0	3	1	?	0
Sangiorgioichthys sui	0	1	0&1	0	0	1	0	0	0	1	0	0	0	1	1	0	1	1	0	2	?	0	0	2	0	0	0	0	1	0	0	2	1	0	0	0	0	0	1	0	0	0	0	0	0	0	1	1	?	?
Sangiorgioichthys aldae	0	1	1	?	0	1	0	0	0	1	0	0	0	1	1	0	1	1	0	2	?	0	?	?	0	?	0	0	?	?	?	2	1	0	0	0	0	0	?	0	0	0	0	0	0	0	1	1	?	?
Luoxiongichthys	1	1	0	?	0	1	0	0	0	1	0	0	0	1	?	?	0	1	0	2	?	?	?	?	0	0	1	0	?	0	0	1	0	0	0	0	?	?	?	?	0	0	0	0	0	0	3	1	?	?
Lophionotus sanjuanensis	0	?	0	?	0	1	0	0	0	2	0	0	0	1	1	0	1	1	1	2	?	1	1	0	0	0	0	1	1	?	?	1	2	0	0	1	0	0	0	0	0	0	0	0	0	0	3	1	?	?
Lophionotus chinleana	0	?	0	?	0	1	0	0	0	1	0	0	0	1	1	0	1	1	1	2	?	0	0	?	0	0	0	1	1	1	0	?	2	0	0	?	0	0	0	0	0	0	0	0	0	0	2	1	?	?
Lophionotus kanabensis	0	1	1	?	0	1	0	0	1	1	0	0	0	1	1	0	1	1	0	2	?	?	?	?	?	?	0	1	?	?	?	?	2	0	0	?	?	0	0	0	0	0	0	0	0	0	2	1	?	?
Camerichthys	0	1	?	0	1	1	0	0	0	1	0	0	0	1	1	0	?	1	0	2	?	?	?	?	?	?	?	?	?	?	?	?	1	0	?	?	?	?	?	?	0	0	?	0	?	1	3	0	?	?
Archaeosemionotus connectens	0	1	0	?	0	0	0	0	0	1	0	0	1	1	1	0	0	1	0	1	?	?	?	?	?	0	0	1	1	?	?	1	?	0	?	?	?	?	?	?	0	0	?	0	0	0	?	?	?	?
Hemicalypterus weiri	0	?	1	?	0	0	0	1	0	1	0	0	0	1	1	0	1	1	1	1	0	0	?	0	?	?	1	0	1	0	0	0	3	0	0	0	?	0	0	0	2	1	1	1	1	0	0	0	?	0&1
Sargodon tomicus	0	?	1	?	1	0	0	1	0	2	1	?	0	1	?	0	0	1	0	?	0	0	0	1	?	?	1	0	1	0	0	?	0	0	1	0	?	?	0	?	1	1	1	1	1	0	0	0	?	0&1
Dandya ovalis	0	?	1	?	0	0	0	0	0	2	?	?	0	1	?	0	1	1	0	1	0	0	?	?	?	0	1	0	1	0	0	?	0	0	?	?	?	?	?	0	0	0	1	1	1	0	?	?	?	0
Dapedium caelatum	0	1	?	0	1	?	0	1	0	2	1	0	0	1	?	0	0	1	0	1	0	0	0	0	0	0	1	0	1	0	0	0	0	0	0	0	?	?	?	0	0	?	0	1	1	0	0	0	?	?
Dapedium stollorum	0	1	0	?	1	0	0	1	0	2	1	0	0	1	1	0	0	1	0	1	?	0	0	0	0	0	1	0	1	0	0	0	0	0	0	0	?	1	?	1	0	0	0	1	1	0	0	0	?	?
Dapedium punctatum	0	?	1	?	1	0	0	1	0	2	1	0	0	0	?	?	0	1	0	1	0	0	0	?	0	0	1	0	1	0	0	0	1	0	0	0	?	?	?	1	0	0	0	1	1	0	0	0	?	?
Dapedium pholidotum	0	1	1	0	1	0	0	1	0	2	1	0	0	0	?	?	0	1	0	1	0	0	0	0	0	0	1	0	1	0	0	0	1	0	0	0	?	?	1	1	0	0	0	1	1	0	0	0	?	?
Paradapedium egertoni	?	?	?	?	0	?	0	?	0	2	1	?	0	0	?	?	1	1	0	?	0	0	0	?	?	0	1	1	?	?	?	?	1	0	0	0	0	?	?	?	0	1	0	1	1	?	?	0	?	?
Tetragonolepis oldhami	?	?	0	?	0	0	?	1	?	1	1	?	0	0	?	?	1	1	0	1	0	?	?	?	?	?	1	0	?	?	?	?	1	0	0	0	?	?	?	?	0	1	0	1	1	?	?	0	?	?
Tetragonolepis semicincta	0	?	0	?	0	0	?	1	0	1	1	0	0	0	?	?	1	1	0	0	?	?	0	?	?	?	1	0	1	0	0	0	1	0	0	0	0	0	0	?	0	1	0	1	1	0	0	0	?	0&1
Aetheolepis	?	?	?	?	?	?	?	?	?	?	1	?	0	?	?	?	?	?	?	?	?	?	?	?	1	1	1	1	0	?	?	0	1	0	0	0	?	?	?	?	?	0	1	1	0	?	?	?	?	0
Kyphosichthys grandei	0	1	0	?	0	0	0	1	0	0	0	1	0	1	1	0	1	1	1	?	?	0	1	?	0	0	0	0	0	0	0	1	1	0	0	?	0	?	?	?	0	0	0	1	0	0	2	1	?	?
Heterostrophus phillipsi	1	?	0	0	?	0	?	1	0	2	1	0	0	0	?	?	1	1	0	1	?	?	0	?	?	?	?	?	1	0	0	0	?	0	0	0	?	?	0	?	0	1	0	1	1	0	3	0	?	?
